# Exploring Taxonomic and Genetic Relationships in the *Pinus mugo* Complex Using Genome Skimming Data

**DOI:** 10.3390/ijms251810178

**Published:** 2024-09-22

**Authors:** Joanna Sikora, Konrad Celiński

**Affiliations:** Department of Genetics, Institute of Experimental Biology, Faculty of Biology, School of Natural Sciences, Adam Mickiewicz University, Uniwersytetu Poznańskiego 6, 61-614 Poznań, Poland; joanna.sikora@amu.edu.pl

**Keywords:** *Pinus mugo* complex, genome skimming, nrDNA, mitogenome, plastome, intergenic spacer, next-generation sequencing

## Abstract

Genome skimming is a novel approach that enables obtaining large-scale genomic information based on high-copy DNA fractions from shallow whole-genome sequencing. The simplicity of this method, low analysis costs, and large amounts of generated data have made it widely used in plant research, including species identification, especially in the case of protected or endangered taxa. This task is particularly difficult in the case of closely related taxa. The *Pinus mugo* complex includes several dozen closely related taxa occurring in the most important mountain ranges in Europe. The taxonomic rank, origin, or distribution of many of these taxa have been debated for years. In this study, we used genome skimming and multilocus DNA barcoding approaches to obtain different sequence data sets and also to determine their genetic diversity and suitability for distinguishing closely related taxa in the *Pinus mugo* complex. We generated seven different data sets, which were then analyzed using three discrimination methods, i.e., tree based, distance based, and assembling species by automatic partitioning. Genetic diversity among populations and taxa was also investigated using haplotype network analysis and principal coordinate analysis. The proposed data set based on divergence hotspots is even twenty-times more variable than the other analyzed sets and improves the phylogenetic resolution of the *Pinus mugo* complex. In light of the obtained results, *Pinus* × *rhaetica* does not belong to the *Pinus mugo* complex and should not be identified with either *Pinus uliginosa* or *Pinus rotundata*. It seems to represent a fixed hybrid or introgressant between *Pinus sylvestris* and *Pinus mugo*. In turn, *Pinus mugo* and *Pinus uncinata* apparently played an important role in the origins of *Pinus uliginosa* and *Pinus rotundata*.

## 1. Introduction

The rapid development of both high-throughput sequencing and advanced bioinformatics methods in recent years, combined with the decreasing costs of performing such analyses, has enabled the widespread use of these advanced methods not only for medical purposes but also in many fields of life sciences, including ecological studies [1,2,3,4,5]. With the use of these powerful tools, it seems that the search for markers to distinguish and identify species has become easier than ever. However, many works show that the use of even the most modern and advanced approaches does not always guarantee an effective solution to seemingly simple but fundamental problems in biological research, such as species identification [6,7,8]. This task remains a major challenge, especially in the case of closely related taxa (so-called complexes), regardless of the development of new and sophisticated research methods.

The *Pinus mugo* complex from the *Pinaceae* family is a large (including 16 species, 91 varieties, and 19 other forms) and taxonomically demanding group of closely related pines found in peat bogs and mountain areas in Central and Southern Europe, i.e., dwarf pine (*Pinus mugo* Turra), peat bog pine (*Pinus uliginosa* Neumann ex Wimmer/*Pinus rotundata* Link), or mountain pine (*Pinus uncinata* Ramond ex DC) [9,10]. Taxa discrimination and identification are problematic in this complex, mainly due to the lack of clarity regarding the nomenclature of individual taxa (the presence of different synonyms in scientific literature, i.e., *Pinus rotundata* as *Pinus uliginosa*) [9,11]; postulated gene flow (both within this complex and with taxa outside it, i.e., *Pinus sylvestris* L. [12,13,14,15,16,17,18]; the formation of interspecific hybrids, characterized by the high morphological and anatomical variability of such individuals (i.e *Pinus* × *rhaetica* Brügger); or complex evolutionary history [19,20]. Although the pine complex is one of the best-studied complexes of closely related taxa, including morphological [21,22,23,24], anatomical [21,25], biochemical [24,26,27,28,29], phytochemical [30,31], as well as genetic and cytogenetic studies [32,33,34,35], the relationships between these taxa are the subject of further research, but their delimitation has remained a major challenge for scientists for many years. The clarification of these complicated relationships is also hampered by the lack of variability at the DNA level observed in selected barcoding regions of chloroplast [36] and nuclear DNA [37].

The latest research conducted on the *Pinus mugo* complex, which involved a comparative analysis of the complete chloroplast genome sequences of three representatives of the *Pinus mugo* complex [38], showed the occurrence of potential genetic differences between them. However, it is difficult to assess their species specificity due to the lack of broader population studies in this area. Undoubtedly, the extension of the analyzed sequences helped to identify these differences and showed that this is the right direction for the analysis of these closely related taxa in future studies.

Genome skimming is a novel approach that allows obtaining large-scale genomic information based on high-copy DNA fractions from shallow whole genome sequencing. This method involves the extraction, assembling, and analysis of genomic DNA fractions present in cells in multiple copies, i.e., nuclear ribosomal cistron (nrDNA), chloroplast DNA (cpDNA), and mitochondrial DNA (mtDNA) [39]. This enables obtaining partial or even complete organellar genomes (plastomes and mitogenomes) as well as large tandem repeats of ribosomal DNA (rDNA) in plants [40]. The indisputable advantage of the method is the generation of sequence sets without the necessity for primer design, PCR amplification, electrophoresis, cloning, or Sanger sequencing. It is notable that genome skimming relies on the analysis of numerous genetic regions, enabling the identification of hotspots and novel, species-specific genetic markers. Given its simplicity and relatively low analysis costs, this approach has been used in many plant studies, including plant systematics, plant identification, DNA barcoding, phylogeny, and even the discovery of new markers [41,42,43]. The method was used also to distinguish species in many taxonomically challenging plant groups, i.e., *Theobroma* [44], *Araucaria* [45], *Diospyros* [6], and *Panax* [46].

The European Mountain pines aggregate provides an ideal model to assess the usefulness of genome skimming and multilocus DNA barcoding approaches for improving phylogenetic resolution and extended barcodes for taxa discrimination. Therefore, in this study, by sampling multiple individuals of different members of the *Pinus mugo* complex and closely related taxa, i.e., *Pinus sylvestris* and *Pinus × rhaetica*, we useda genome skimming approach coupled with bioinformatic and statistic pipelines to: (1) obtain large genomic data sets representing the most abundant mitochondrial, chloroplast, and nuclear regions of the genome; (2) generate diverse sequence data sets and determine their genetic variability and usefulness in studies of closely related pine taxa; (3) determine the effectiveness of discriminating between analyzed taxa using different data sets and delimitation methods; (4) reveal the genetic relationships of *P. × rhaetica*, *P. rotundata*, and *P. ulignosa* to each other; and (5) screen the genetic diversity of taxa and populations of the *Pinus mugo* complex.

The results of our study not only provide new and extensive genomic resources for further studies on the *Pinus mugo* complex, but also allow us to answer the question about the effectiveness and validity of using the genome skimming approach to perform analyses of other complexes of closely related taxa.

## 2. Results

### 2.1. DNA Sequencing, Assembly, and Annotation

Ion Torrent sequencing generated between 5,846,195 and 10,076,095 reads per sample. In samples, fifty-seven complete chloroplast genes, eighteen plastid intergenic spacer regions, and fifteen complete mitochondrial coding genes were identified using the genome skimming approach and bioinformatics methods. Additionally, a complete nrDNA cistron was also obtained, and this is the first report of it in representatives of the *Pinus mugo* complex. The total length of the nrDNA sequence was 7848 bp. The lengths of the individual parts were 1812 bp, 2482 bp, 158 bp, 241 bp, and 3155 bp for 18S, ITS1, 5.8S, ITS2, and 26S, respectively. A set of five divergent regions, selected on the basis of their significantly higher-than-average level of genetic variability, was also analyzed. A list of analyzed sequences and accession numbers is provided in the Supplementary Materials (Tables S2, S7–S10).

### 2.2. Genetic Characterization of Diversity in Data Sets

In this study, seven different genetic data sets were analyzed, namely: plastid coding genes (A), mitochondrial coding genes (B), plastid intergenic regions (C), nrDNA cistron (D), ITS (E), *matK* + *rbcL* (F), and divergence hotspot regions (G). In each data set, all sequences were concatenated, and then we determined the alignment length, variable sites, and parsimony informative sites, as well as percentage of variable sites and parsimony informative sites. For an easier comparison, these parameters are presented in two separate tables. Table 1 includes the results for taxa from the *Pinus mugo* complex. Table S3 includes the results obtained for *Pinus* × *rhaetica* and *Pinus sylvestris*. Seven data sets differed in all the above-mentioned parameters. For the *Pinus mugo* complex, the longest data set obtained was set A (45,467 bp) and the shortest was set G (1021 bp). The number of variable sites varied between data sets from three for set E to 128 for set B. The highest number of informative parsimony sites was found for set B (68) and the lowest (0) for data set E. The highest percentage of variable sites was found for set G (1.959%) and it was almost twenty-times higher than that observed in set E (0.104%), which was the least variable in this parameter. The percentage of informative parsimony sites ranged from 0% for set E to 1.469% in set G. For *Pinus × rhaetica* and *Pinus sylvestris*, similar results were obtained to those observed for the *Pinus mugo* complex. The longest data set was set A (45,467 bp) and the shortest set G (1021 bp).

The number of variable sites varied between data sets from 0 for sets E and F to 33 for set B. There were no informative parsimony sites for *Pinus × rhaetica* and *Pinus sylvestris* in the tree data sets analyzed (sets D, E, and F) (Table S3). The level of genetic variation observed in *Pinus × rhaetica* and *Pinus sylvestris* was lower than that observed for the *Pinus mugo* complex. Overall, data set B is characterized by the largest number of variable sites in both the *Pinus mugo* complex and *Pinus × rhaetica* + *Pinus sylvestris*. In percentage terms, the G data set is the most variable in both cases considered.

The order of the data sets from most to least variable in percentage terms for the *Pinus mugo* complex taxa is G > B > C > F > A > D > E.

### 2.3. Delimitation of Taxa

#### 2.3.1. Tree-Based Method

In the phylogenetic analyses, a species was deemed successfully identified when all individuals of the same species formed a monophyletic group with a support value exceeding 50%. The results of the tree-based analyses performed with different input data sets were not fully consistent or unambiguous.

When considering individual data sets, it can be seen that data set A, although the longest in terms of the base pair, did not allow for the successful identification of the analyzed taxa, and the discrimination success using this data set was zero. The mitochondrial coding genes (data set B) showed higher discriminatory power than plastid coding genes (data set A) because two taxa could be distinguished, i.e., *Pinus sylvestris* and *Pinus uncinata*. The remaining taxa did not form highly supported monophyletic groups. The data set covering the plastid intergenic regions (data set C) allowed us to distinguish three taxa, while set D identified only one taxon (Table 2). Data sets E and F did not extract any taxon. The highest success of taxonomic discrimination was characterized by set G. Using divergence hotspots, three taxa can be distinguished.

**Figure 1:**
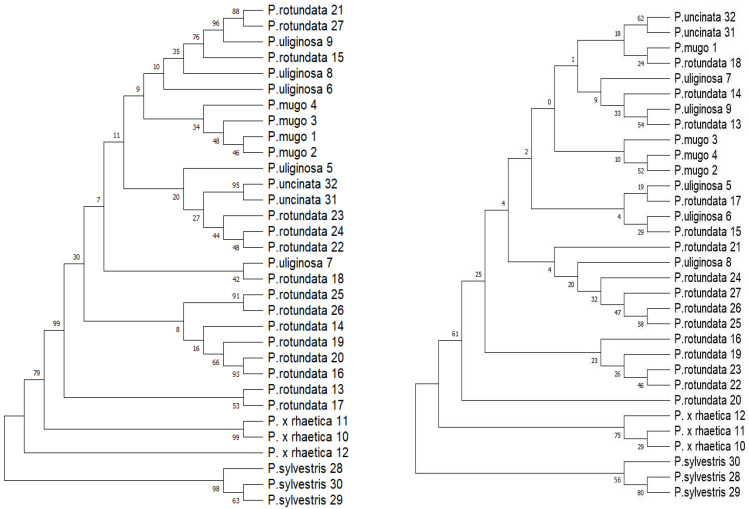
Maximum likelihood trees based on the plastid intergenic regions data set C (**left**) and divergence hotspot regions data set G (**right**).

Considering individual taxon in turn, *P. mugo*, *P. uncinata*, *P. uliginosa*, *P. rotundata* from the *Pinus mugo* complex, as well as *P. × rhaetica* and *P. sylvestris* cannot be distinguished using A, E, and F data sets (Figures S1, S4 and S5). All *Pinus uncinata* samples were considered monophyletic and distinguished from other taxa of the *Pinus mugo* complex using data sets B, C, D, and G with 63 to 95% bootstrap support (Figure 1 and Figures S2 and S3). Similarly, all *Pinus sylvestris* individuals formed a monophyletic clade in data sets B, C, and G with 56% to 98% bootstrap support. All *Pinus mugo* specimens were resolved as monophyletic only with data set C (34% bootstrap support), and *P. × rhaetica* with data set G (75% bootstrap support) (Figure 1). *Pinus uliginosa* and *Pinus rotundata* individuals were not resolved as monophyletic for either the ITS, the broader nrDNA, or any of the plastid and mitochondrial data sets. In turn, *P. × rhaetica* was placed on the maximum likelihood phylogenetic tree between *Pinus sylvestris* individuals and taxa from the *Pinus mugo* complex based on data set C, and in data set G in the same clade as *Pinus sylvestris* (Figure 1).

#### 2.3.2. Distance-Based Methods

In the distance analyses, a species was deemed successfully identified when its minimum interspecific distance exceeded its maximum intraspecific distance. The distance-based method exhibited a trend similar to that of the tree-based method. 

Seven data sets (A–G) differed in their success in discriminating taxa. Sets A, E, and F did not differentiate taxa at all, while for sets D and B, C distinguished one (D) and two taxa (B,C), respectively. The highest discriminatory success (50%) was provided by data set G. It was possible to distinguish three taxa out of six analyzed. In data set G, the highest values were observed for both interspecific and intraspecific distances, 0.00295 and 0.01285, respectively (Table S4).

Considering individual taxa, for *Pinus mugo*, *Pinus rotundata*, and *Pinus uliginosa*, in each data set, their maximum value of intraspecific variability exceeded the minimum interspecific variability, making them impossible to delimit (Table S4). For *Pinus uncinata*, the minimum interspecific value was higher than the maximum intraspecific value in four of the seven data sets, i.e., B, C, D, and G, thus allowing the delimitation of this taxon. *Pinus × rhaetica* can be distinguished using three data sets, i.e., B, C, and G, while *Pinus sylvestris* can be distinguished only using data set G (Table S4).

#### 2.3.3. Assembling Species by the Automatic Partitioning Method (ASAP)

**Figure 2:**
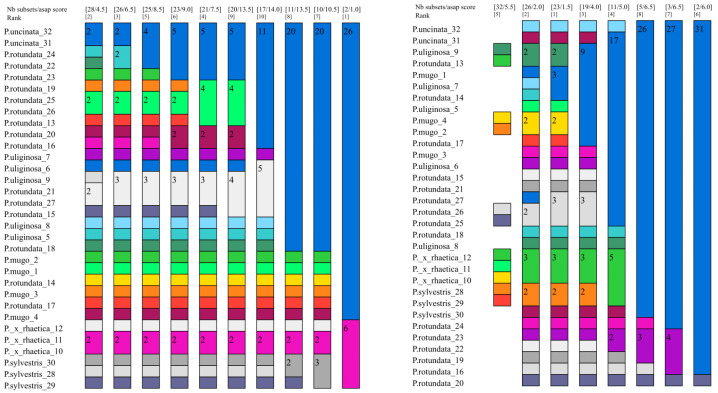
Species delimitation by ASAP analysis for two data sets: the plastid intergenic regions (**left**) and divergence hotspot regions (**right**). Graphical output showing the ten different delimitations: each column represents a partition, and the colors represent the operational taxonomic units (OTUs). Every field contains the number of individuals. Above the colorful bars, the coefficient asap-score (the lower value) and number of species (the upper value) recognized for whole data sets are presented.

In the case of the ASAP method, the obtained results differed significantly depending on the input data set used (Figure 2 and Figures S6–S10). For the plastid data (set A), ASAP indicates the presence of three or two partitions. In the case of two partitions, all *Pinus sylvestris* and *Pinus × rhaetica* individuals are in one partition and the rest of the individuals constitute the second one, corresponding as it were to the *Pinus mugo* complex. In the case of mitochondrial data, we observe a large fragmentation in the range of the partitions. There are 27 of them in total and they include mostly single individuals. One partition includes six individuals and contains representatives of *Pinus uliginosa, Pinus mugo*, and *Pinus × rhaetica*—although these are not all analyzed individuals from these taxa. The rest of the individuals representing these taxa occur in separate partitions. Set C (plastid intergenic regions) is basically consistent with set A. We observe two partitions: in one, 26 individuals of the *Pinus mugo* complex and, in the other, six individuals—all analyzed individuals of *Pinus sylvestris* and *Pinus × rhaetica*. The cistron data show four partitions: one containing 29 individuals and three smaller partitions containing single individuals—each consisting of representatives of a different taxon. The ASAP score for the ITS data set is similar to the results obtained for cistron. Two partitions were distinguished: one has 31 individuals and the other only one. In terms of the barcode sequences (data set F), the lowest ASAP score was found for four partitions. The first partition contains 24 individuals from different taxa included in the *Pinus mugo* complex. The second largest partition contains six individuals, and these are all representatives of *Pinus sylvestris* and *Pinus × rhaetica*. Partitions three and four contain one individual each of *Pinus rotundata* (sample no. 27 from the Czech Republic) and *P. rotundata* (sample no. 21 from Germany). The divergence hotspots data indicate the presence of 23 partitions. Among them, three partitions contained three individuals each. The most homogeneous partition was the one composed exclusively of *Pinus × rhaetica* from “Bór na Czerwonem” (Poland) and exclusively of *Pinus rotundata* from the “Červené blato” population (Czech Republic). The third three-individual partition was already mixed and contained representatives of *P. uliginosa*, *P. mugo*, and *P. rotundata*.

In summary, the results of the taxon delimitation using three different approaches differ significantly from each other. The highest level of success in distinguishing taxa was achieved using the distance-based method and the G data set. The ASAP method had the lowest success rate, regardless of the input data used (Table 2).

### 2.4. Genetic Relationships of Taxa and Populations

**Figure 3:**
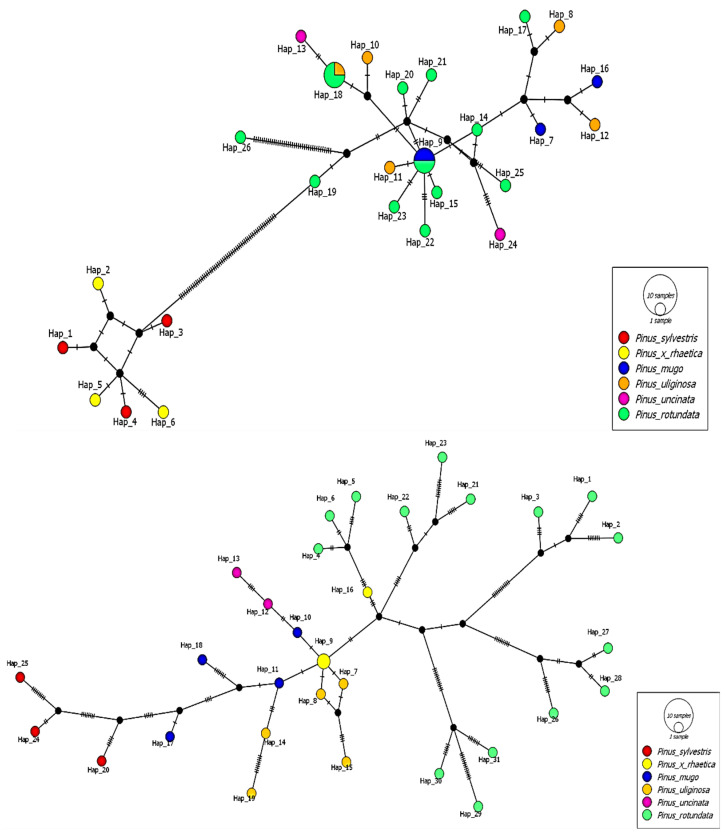
Median-joining haplotype network of the 32 individuals analyzed based on chloroplast protein coding genes (data set A) (**upper**) and based on mitochondrial protein coding genes (data set B) (**lower**).

Having a set of chloroplast data (set A) and mitochondrial data (set B), we determined the mutual relationships between the analyzed taxa using a median-joining haplotype network (Figure 3) constructed using PopArt [47]. Particular emphasis was placed on *Pinus* × *rhaetica* because the results obtained from taxon delimitation showed interesting trends.

This analysis revealed 26 chloroplast haplotypes (chlorotypes). Haplotype 9 is shared by two individuals of *Pinus mugo* and *Pinus rotundata*. Haplotype 18 is common to one individual of *Pinus uliginosa* and three individuals of *Pinus rotundata*. The highest genetic distance expressed in the number of nucleotide differences was observed between *Pinus* × *rhaetica* and *Pinus sylvestris* and the rest of the individuals belonging to the *Pinus mugo* complex. The analysis of the relationship of the *Pinus mugo* complex, *Pinus* × *rhaetica*, and *Pinus sylvestris* based on complete mitochondrial genes (data set B) revealed the existence of 31 haplotypes (mitotypes). Two individuals of *Pinus* × *rhaetica* have the same haplotype (haplotype 9). Interestingly, all mitochondrial haplotypes of *Pinus rotundata*, *Pinus uliginosa*, and *Pinus uncinata* corresponded to the population from which the individuals were collected. The list of detected chloro- and mitotypes is provided in Tables S5 and S6.

**Figure 4:**
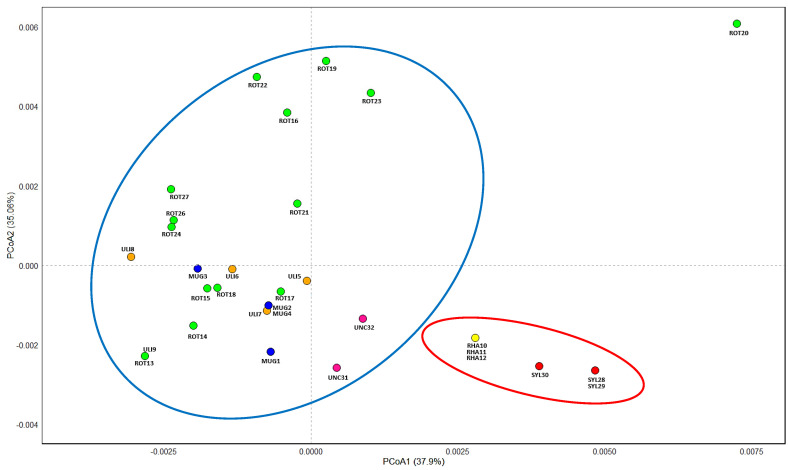
Results of PCoA analysis based on divergence hotspot regions. Abbreviations: MUG—*Pinus mugo*; UNC—*Pinus uncinata*; ULI—*Pinus uliginosa*; ROT—*Pinus rotundata*; RHA—*Pinus × rhaetica*; SYL—*Pinus sylvestris*.

Principal coordinate analysis (PCoA) performed on the divergence hotspots regions clearly distinguished taxa belonging to the *Pinus mugo* complex (larger group) from *Pinus sylvestris* and *Pinus × rhaetica* (smaller group) (Figure 4). Within each of these groups, two smaller subgroups can also be distinguished. Within this larger group, individuals of *Pinus uncinata* clearly stand out, while individuals of *Pinus mugo*, *Pinus rotundata*, and *Pinus uliginosa* form an extensive cloud. In a smaller group, differences between *Pinus sylvestris* and *Pinus × rhaetica* are visible, the latter being clearly closer to *Pinus sylvestris* than to taxa from the *Pinus mugo* complex.

**Figure 5:**
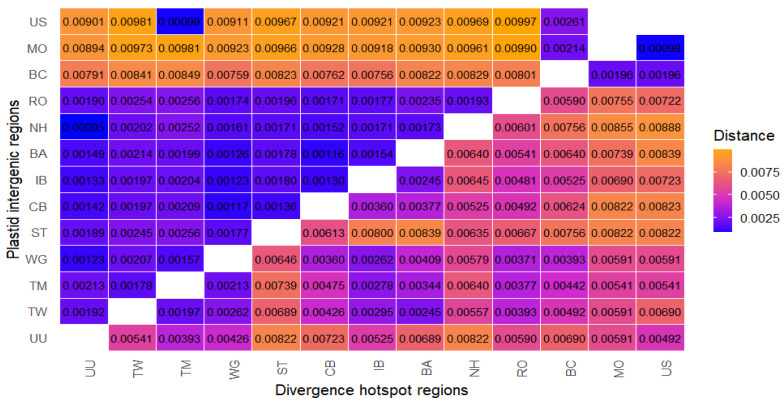
Heat map for pairwise genetic distances between populations. K2P genetic distance matrix on the plastid intergenic and divergence hotspot regions of 13 samples of the *Pinus mugo* complex, *Pinus × rhaetica*, and *Pinus sylvestris* taxa (the lower half of genetic distances belong to divergence hotspot regions and the upper half of distances to the plastid intergenic regions). Genetic distance is low to moderate: 0.0025 < x < 0.0050 (blue to purple); high: 0.0050 < x < 0.0075 (fandango to orange).

The mean nucleotide divergence (Kimura 2-parameter) among the 13 samples ranged from 0.00095 to 0.00997 for the plastid intergenic region (data set C) (Figure 5). The largest genetic distance occurs between the *Pinus rotundata* from the “Rotmeer” Nature Reserve (Germany) and *Pinus sylvestris* from the Dendrological Garden, University of Life Sciences (US) (Poland). Conversely, the smallest genetic distance was observed between the Polish population of *Pinus uncinata* (US—Dendrological Garden, University of Life Sciences) and *Pinus rotundata* from the Czech Republic (NH—“Novohůrecká slat”). For divergence hotspot regions (data set G), the genetic distances range from 0.000981 to 0.008882 (Figure 5). The largest genetic distance occurs between the *P. rotundata* population from “Novohůrecká slat” (NH) (Czech Republic) and *Pinus sylvestris* from the Dendrological Garden, University of Life Sciences (US) (Poland). The smallest genetic distance is between the Polish populations of *Pinus sylvestris* (US—Dendrological Garden, University of Life Sciences and MO—Morasko, Poznań).

## 3. Discussion

The aims of this work were to generate a large amount of genomic data from multiple representatives of particular closely related taxa based on the genome skimming method and use them to characterize the genetic variability present in different data sets. We paid special attention to searching for a genetic data set in which one could try to identify interspecific differences, improve the phylogenetic resolution, and also describe the overall genetic variability present within individual populations and taxa. This task was a major challenge because previous attempts to find species-specific DNA markers for *Pinus mugo, Pinus uliginosa*, and *Pinus uncinata* based on eight selected chloroplast DNA barcoding regions, including traditional core barcodes (*matK + rbcL*), candidate (*trnH-psbA*), or even hypervariable regions (*ycf1* and *ycf2*) [36], showed that, in the case of these closely related taxa from the taxa of the *Pinus mugo* complex, these regions are not variable and there are no interspecific differences (at least in the analyzed area). Increasing the search area for differences from over five-thousand nucleotides (the work mentioned above) to almost one-hundred-and-twenty-thousand nucleotides, namely in the form of complete chloroplast genomes [38,48], allowed us to identify some potential species differences in a few regions. However, it is difficult to consider them as fully differentiating taxa because they were not tested on a larger number of individuals. Due to the complexity of the relationships in the *Pinus mugo* complex, there are few studies examining more than a few taxa in a multi-population system [19,49]. Unfortunately, without such a broader sample and knowledge of intraspecific variability, it is difficult to both search for and verify potential species-specific markers. Therefore, in this work, we focused on the analysis of many individuals from a given taxon in order to also determine the level of intraspecific variability and be able to compare it with the level of interspecific variability (and thus determine, among other things, the barcoding gap). We also wanted to understand the variability and relationships not only at the species level, but also at the population level.

Genome skimming is a shallow whole-genome sequencing technique that usually provides enough coverage for a complete assembly. In plants, this method often yields adequate coverage of high-copy regions, enabling the recovery of complete or partial organellar genomes and nuclear ribosomal DNA sequences. This method was used, among others, to recover the complete plastomes of *Theobroma* spp. [44], *Fargesia* [50], *Paris* [51], *Rhododendron* [52], *Panax* [46], *Areca* [53], *Piper*, as well as *Peperomia* [54] and *Salix* [55]. Numerous publications have highlighted the value of data obtained through genome skimming in relation to the valuable genomic resources archived in museums and herbaria [56,57]. Nevertheless, in complex plant groups like *Panax* [46] and *Araucaria* [45], the plastome and nrDNA data did not significantly enhance the discriminatory power. The recent diversification of several lineages of the *Panax bipinnatifidus* species complex may result in plant barcodes being shared with closely related species and thus not delimiting species boundaries [44].

Genome skimming proved to be extremely helpful in our studies, as it allowed us to obtain many very long regions representing the genomes of different organelles and to generate seven data covering: plastid coding genes; mitochondrial coding genes; plastid intergenic regions; nrDNA cistron; ITS; *matK* + *rbcL*; and divergence hotspot regions. The same method was recently used to generate sequence data also for *Cymbidium* [8] and *Rhododendron* [52]. In these studies, genetic diversity and species delimitation ability were analyzed using different combinations of DNA regions, including complete plastomes (superbarcoding approach), plastomes + nrDNA (ultrabarcoding approach), a mixture of barcoding regions in a narrower sense, i.e., nrDNA, ITS, *rbcL*, *matK*, *trnH-psbA* (core DNA barcode regions), and, in a broader sense: *matK* + *rbcL* + *trnH-psbA*, ITS + *matK* + *trnH-psbA*, and ITS + *matK* + *rbcL* + *trnH-psbA*. The level of observed genetic variation measured by the percentage of divergence was highly variable and differed significantly within individual taxa and regions. In *Rhododedron* species, the most variable region was the ITS region (17.05% divergence) before the combination of ITS + *matK* + *trnH-psbA* (15.88%) [52]. In turn, in *Cymbidium* species [8], the most variable region was the *matK* region (16.65% divergence) before the combination of *rbcL* + *matK* + *trnH-psbA* (8.27% divergence). In *Panax* [46] and *Fargesia* [50] species, the ITS region was also the most variable (12.25% and 10.60% divergence values, respectively). It is worth noting that the length of the ITS region in all the species mentioned above is about 600 to 700 nt. These results confirm that the ITS is a very good candidate for an interspecific marker, at least in these plants.

The analysis of the ITS region in conifers has been, to date, significantly hampered by the size of the nuclear genome [58,59,60] and the length and complexity of the sequence itself [61,62]. Therefore, there are not many works using this region in the analyses of conifers [62,63,64,65]. However, the use of genome skimming in this study allowed us to determine and characterize for the first time the complete ITS and nrDNA sequence for representatives of the *Pinus mugo* complex.

Our results contradict previous literature reports of high ITS polymorphism in some species [46,50,52]. Both ITS and the whole nrDNA in the case of the analysis of taxa in the *Pinus mugo* complex were the regions with the lowest level of variability (0.104% and 0.127%, respectively) of all the data sets we analyzed. This is despite the fact that the ITS and nrDNA regions were significantly longer in the *Pinus mugo* complex representatives than in other taxa. The length of the ITS in the *Pinus mugo* complex was 2881 nucleotides, and the length of the nrDNA was 7849 nucleotides. Previous studies of ITS2, which is ten-times shorter than ITS1 [37], showed that ITS2 is quite good for identifying taxa in the *Pinaceae* family, but only for some genera. In the case of the *Pinus* genus and the *Pinus mugo* complex, this was not particularly the case. This region was simply too short and showed too little variability in this group of plants. It was therefore suggested that perhaps ITS1, as a longer fragment, would be better in this respect. Our results clearly indicate that neither ITS1 nor ITS2 is sufficiently variable to be used for the analysis and differentiation of closely related taxa in the *Pinus mugo* complex. Despite our observations, ITS2 still remains a very good, or even ideal, barcode for many other plant groups than the *Pinus* genus, as it has many advantages [50,66,67].

Compared to ITS, the nrDNA sequence had a slightly higher discrimination rate, probably due to the larger number of parsimony information sites. However, neither ITS nor nrDNA was able to distinguish all taxa. It therefore seems reasonable to continue searching for other nuclear regions. Finding them would certainly be very helpful for identifying hybrid individuals. Perhaps a good direction is the analysis of single copy genes. Genome skimming can be difficult to use for this purpose because only well-represented fragments are sequenced in this approach and there is not much pure nuclear DNA in the sample. Probably with very high genome coverage there would be a chance to capture single copy genes. Many studies indicate that single gene copies are an interesting tool [68,69].

Chloroplast core DNA coding regions (*matK* + *rbcL*) analyzed in this work as set F, similarly to ITS and nrDNA, were characterized by a low level of variability (0.269% divergence). Using a combination of these regions did not allow us to distinguish closely related taxa from the *Pinus mugo* complex, and this was an expected result after our previous analyses conducted in 2017 [36]. The reasons why these taxa cannot be distinguished using these DNA markers may be many, ranging from incomplete lineage sorting (ILS) to gene exchange (hybridization) or recent reticulate divergence/evolution [19]. In the *Pinaceae* family, chloroplast DNA is assumed to be inherited from the paternal line, although recent studies shed a slightly different light on this [70], which may also have influenced our results.

Our results show that intergenic plastid and hotspot divergence regions (*rps1*, *rps2*, *rps14*, *ycf3-psaA*, and *trnE-clpP*) discriminate between 33.3% and 50.0% of the analyzed taxa using the tree-based and distance-based methods. Hotspot divergence regions, despite having the shortest alignment length, had the highest evolutionary rate of all data sets. This was the only data set in the ASAP method that was able to discriminate any species. In contrast, mitochondrial coding genes showed greater discrimination power than plastid coding genes, up to tenfold. Not surprisingly, the phylogenetic trees obtained from these data differ significantly. According to Wang [71], these discrepancies suggest a series of mtDNA capture events during past range shifts of pine species, indicating that vertical and horizontal inheritance have played a role in the evolution of mtDNA in *Pinus*.

Taxonomists have been using molecular methods for many years to differentiate species more effectively. DNA barcoding in this regard is still a valuable method. However, over time, instead of one or two core barcode regions, researchers have begun to analyze several or a dozen of them (multilocus DNA barcoding) to improve the efficiency of delimitation. Sometimes, a complete chloroplast genome sequence is also very helpful, proposed as a so-called superbarcode [72]. Recently, very satisfactory results have been obtained by using ultrabarcoding—a promising approach that solves many challenges associated with currently common DNA barcoding methods. Ultrabarcoding involves expanding from traditional DNA barcodes to entire plastomes and nuclear ribosomal DNA (nrDNA) sequences [44]. This approach generates a significant amount of data and, as a result, can provide information necessary to investigate variations below the species level. In the case of the *Pinus mugo* complex, ultrabarcoding is unlikely to be useful because nrDNA has poor discriminatory power. Although, of course, there are studies on other plant species where this approach is effective [44,51].

The correct identification and differentiation of species are of great importance not only for purely cognitive or scientific reasons, but also for practical reasons. Problems with the unambiguous identification of protected or endangered taxa translate directly into problems with the preparation, undertaking, and implementation of appropriate protective measures. Closely related taxa, which are characterized by very similar morphologies and a lack of clear species determinants, are usually grouped into larger taxonomic units called complexes. They also pose a great challenge for researchers due to the complex or unknown origin of individual taxa and the presence of many different synonymous names in the literature. The *Pinus mugo* complex is one of many such complexes, but several others are known and this is in the genus *Pinus* itself, e.g., [10,73,74]. Additionally, in the case of sympatric populations of closely related taxa, hybridization and introgression processes occur. The resulting hybrids and introgressants representing an intermediate phenotype between the habit of the parent taxa make it even more difficult to correctly identify individuals from individual taxa. Hence, it is so important to develop appropriate methods and markers that would facilitate the reliable and unambiguous identification of individuals, including hybrids, regardless of the morphological determinants that depend on environmental conditions or require a specific developmental phase.

The *Pinus mugo* complex includes closely related taxa that occur in the most important mountain ranges in Europe, such as the Pyrenees, the Alps, and the Carpathians [9]. These taxa fulfill very important ecological functions and are the basis of many mountain ecosystems. At the same time, discussions have been ongoing for many years on the taxonomic status, origin, and final number of taxa included in this complex. Some researchers distinguish one main species, *Pinus mugo*, with two subspecies, *Pinus mugo* ssb. *mugo* and *Pinus mugo* ssb. *uncinata*, which occur in the mountains of Central and Eastern Europe and Western Europe, respectively [9]. Others, however, distinguish three main species in this complex, i.e., *Pinus mugo*, *Pinus uncinata*, and *Pinus uliginosa*. The latter taxon—*Pinus uliginosa*—is one of the most enigmatic and controversial taxa from the *Pinus mugo* complex [48]. This pine was first described at a site in Wielki Torfowisko Batorowskie in Poland in 1837 [75]. In Poland, this species represents one of the four native species of pines (apart from Scots pine (*Pinus sylvestris* L.), dwarf mountain pine (*Pinus mugo*), and Swiss pine (*Pinus cembra*)) and occurs only at four sites, mainly peat bogs or wetlands. It is a protected species [76] and is threatened with extinction. The most serious threat to the continuity of this species is primarily the poor health condition of the trees, poor natural regeneration, and the risk of contamination of the gene pool of swamp pines due to the uncontrolled gene exchange with Scots pine and dwarf pine that occurs at sympatric sites [12,14].

*Pinus uliginosa*, *mugo*, and *uncinata* are characterized by high morphological variability on the one hand and genetic invariability on the other hand, which does not allow for their effective and unambiguous distinction [36]. Another problem is the identification of individuals of the protected peat bog pine and its postulated hybrids with dwarf mountain pine. Effective protection of the peat bog pine in Poland is additionally complicated by its legal status, because in the Regulation of the Minister of the Environment of October 9, 2014, the peat bog pine is listed under the name *Pinus* × *rhaetica* [76]. It is not clear whether the name *Pinus* × *rhaetica*, which refers to peat bog pine, is identical to the name *Pinus uliginosa*. The relationship between *Pinus uliginosa* and *Pinus* × *rhaetica* remains unclear. It is also not known whether *Pinus uliginosa* is a separate species or, as some researchers postulate, a hybrid of Scots pine (*Pinus sylvestris* L.) with dwarf mountain pine (*Pinus mugo* Turra (as *Pinus* × *rhaetica*)) or a hybrid of mountain pine (*Pinus uncinata* Rammond) with dwarf mountain pine (*Pinus mugo* Turra (*Pinus uncinata* × *Pinus mugo*)) [9]. The results of our research rather confirm the latter hypothesis. Moreover, the results of the research on Polish populations of the peat bog pine—*Pinus uliginosa* or *Pinus* × *rhaetica*—are difficult to directly relate to the results of the research from abroad, especially from the Czech Republic and Germany, because, in the scientific literature, peat bog pine is listed under the name *Pinus rotundata* [11,77]. Therefore, one of the goals of this work was to conduct a phylogenetic inference to confirm or exclude the hypothesis that *Pinus uliginosa* from Poland is the genetic equivalent of *Pinus rotundata* from the Czech Republic and Germany, and both names are synonyms referring to the same taxon.

There are many more questions regarding *Pinus uliginosa*/*Pinus rotundata*, e.g., did it arise independently as a hybrid at different times and in different places (peat bogs in Poland, Germany, and the Czech Republic), giving rise to a taxon with a similar habit and genetic background, due to the similar parental composition? Perhaps it arose in one place and then colonized further regions. Or, maybe, the currently observed populations are relicts and represent the remains of an ancestral species with a wider range. To answer these questions, it is necessary to conduct further research with geographically broader sampling.

The data obtained in this study indicate that *Pinus* × *rhaetica* is not identical to *Pinus uliginosa* or any other analyzed taxon belonging to the *Pinus mugo* complex. These results are in full accordance with the previously conducted research on *Pinus mugo* complex taxa discrimination using seed storage protein profiling [29]. Celiński et al. (2020) analyzed the patterns of seed total proteins and showed that each of the closely related taxon from this complex has a different and unique protein pattern. Moreover, based on the results obtained, *Pinus* × *rhaetica* is much closer to *Pinus sylvestris* than to *Pinus mugo* and *Pinus uliginosa* from the *Pinus mugo* complex. The distinctiveness of *Pinus* × *rhaetica* has also been demonstrated previously in cytological studies using C-banding methods and flow cytometric analysis [33]. This study showed that three closely related pines from the *Pinus mugo* complex (*Pinus mugo*, *Pinus uliginosa*, and *Pinus* × *rhaetica*) differ in some karyotypical features and DNA content. Celiński et al. (2019) therefore postulated not to use the names *Pinus* × *rhaetica* and *Pinus uliginosa* interchangeably as synonyms because these taxa differ from each other.

Our work also presents new data on the genetic diversity of bog pines from the Czech Republic and Germany. Knowledge regarding this subject is still relatively scarce because, in many studies on the *Pinus mugo* complex, *Pinus rotundata* is omitted, and researchers focus mainly on the *Pinus mugo*–*Pinus uncinata* system.

The revealed pattern of genetic variability based on the mitochondrial genome clearly indicates the very unique, local, and isolated character of the analyzed populations from the *Pinus mugo* complex. Attention is paid to both the peripheral location of the Czech and German populations of *Pinus rotundata* and the fact that the Polish populations of *Pinus uliginosa* show a different character. Such a result may indicate the relatively long spatial isolation and the complex historical background of individual populations, especially taking into account the phylogeographic structure of *Pinus mugo* or *Pinus uncinata* as putative parent taxa of *Pinus rotundata* and *Pinus ulignosa* [20].

## 4. Materials and Methods

### 4.1. Plant Material and DNA Extraction

**Figure 6:**
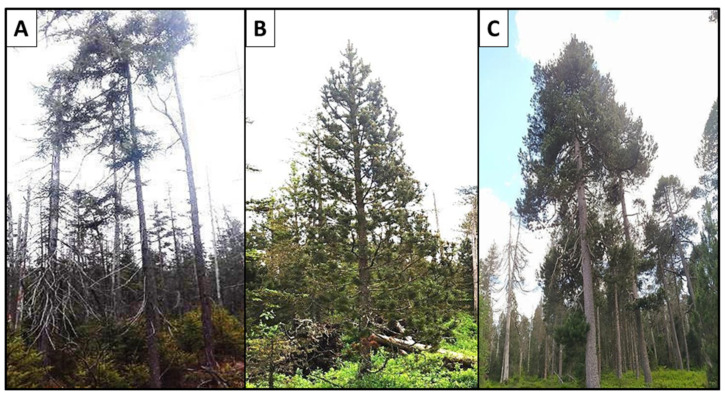
The habitats of selected taxa from the *Pinus mugo* complex. (**A**) *Pinus uliginosa*—“Wielkie Torfowisko Batorowskie” Nature Reserve (BA), Poland; (**B**) *Pinus rotundata*—“Novohůrecká slat” (NH), the Czech Republic; (**C**) *Pinus rotundata*—“Kirchspielwald-Ibacher Moos” Nature and Forest Reserve (IB), Germany.

In order to best represent the *Pinus mugo* complex, four taxa from the *Pinus mugo* complex were analyzed: *P. mugo, P. uncinata, P. uliginosa*, and *P. rotundata*. Each of these taxa was represented by several individuals from one to five populations. Three *Pinus sylvestris* and three *Pinus × rhaetica* individuals were also included in this study as the taxa most closely related to the *Pinus mugo* complex. A total of 32 pine-needle samples was sequenced. Details of individual specimens are presented in Table S1 and the habitats of selected taxa from the *Pinus mugo* complex are presented in Figure 6. Vouchers of the analyzed individuals were deposited in the Natural History Collections, Faculty of Biology, Adam Mickiewicz University, Poznań, Poland (Table S1).

Genomic DNA extraction was performed from 100 mg of fresh plant tissue (needles) using the Genomic Mini AX Plant Spin Kit (A&A Biotechnology, Gdynia, Poland). Sample DNA quality was assessed using the Agilent High-Sensitivity D1000 ScreenTape System (Agilent Technologies, Inc., Waldbronn, Germany). Until analysis, the isolated DNA was stored in a freezer at −20 °C.

### 4.2. DNA Sequencing, Assembly, and Annotation

Ion Torrent barcoded libraries with approximately 300 bp insert fragments were constructed using the Ion Xpress™ Plus Fragment Library Kit and Ion Xpress™ Barcode Adapters Kit (ThermoFisher Scientific, Waltham, MA, USA). Libraries were sequenced using the Ion GeneStudio™ S5 System (Thermo Fisher Scientific, Waltham, MA, USA). Adapters and low-quality reads were filtered using BBDuk Adapter/Quality Trimming V. 38.84 by Brian Bushnell implemented in Geneious Prime 2020.2.5 [78].

A genome skimming method was used to generate nucleotide sequences from the chloroplast, mitochondrial, and nuclear genomes for *Pinus mugo* complex taxa, as well as *Pinus sylvestris* and *Pinus × rhaetica* individuals. For this purpose, reads were assembled de novo using Geneious Assembler on default settings. Resulting contigs were mapped to the reference chloroplast genome of *Pinus sylvestris* (NC_035069.1), *Pinus taeda* mitochondrion (NC039746), and *Pinus sylvestris* nrDNA cistron (MT735327) using Bowtie2 v.2.4.5 [79]. Then, the complete protein coding genes and intergenic spacer regions were extracted from the resulting incomplete chloroplast genomes using Geneious Prime 2020.2.5 [78]. The internal transcribed spacer sequences (ITS1, 5.8S, and ITS2) were extracted from nrDNA cistron in Geneious Prime 2020.2.5 [78].

### 4.3. Data Analysis

#### 4.3.1. Genetic Characterization of Diversity in Data Sets

We constructed seven DNA data sets by concatenating different aligned regions, including: (A) the concatenated plastid coding genes; (B) the concatenated mitochondrial coding genes; (C) the plastid intergenic regions; (D) the complete nuclear ribosomal DNA (nrDNA); (E) the internal transcribed spacer (ITS) consisting of ITS1-5.8S-ITS2 sequences; (F) core barcode regions *matK + rbcL*; and (G) divergence hotspot regions (*rps1*, *rps2*, *rps14*, *ycf3-psaA*, and *trnE-clpP*). The alignment of each region was conducted using MAFFT v7.490 [80,81]. MEGA11 [82] was used to calculate the alignment length, number of variables, and parsimony informative sites, as well as the percentage of variable sites and percentage of parsimony informative sites among representatives of the *Pinus mugo* complex, *Pinus × rhaetica*, and *Pinus sylvestris*. The diversity thresholds, used to select variable regions, were calculated by the sum of the average and double the standard deviation [83].

#### 4.3.2. Delimitation of Taxa

To evaluate the effectiveness of taxa discrimination, we performed analyses based on the created seven data sets using three different delimitation methods. The tree-based method was performed using the maximum likelihood method with the option of 1000 bootstrap replicates conducted in MEGA 11 [82]. To estimate the best nucleotide substitution model for each data set, the “Find Best Fit Substitution Model” option available in MEGA11 was used [84]. Phylogenetic trees were created for the 26 analyzed individuals belonging to the *Pinus mugo* complex, 3 individuals of *Pinus × rhaetica*, and 3 individuals of *Pinus sylvestris*. The distance-based method was performed using the pairwise distance calculated in MEGA 11 [82] using the Kimura 2-parameter (K2P) model. The taxa with the larger minimum interspecific distance than the maximum intraspecific distance were consider as successfully identified. We also used the ASAP (Assemble Species by Automatic Partitioning) web server available at https://bioinfo.mnhn.fr/abi/public/asap. The ASAP analysis was conducted using the Kimura K80 substitution model (ts/tv = 2.0). ASAP employs automatic partitioning algorithms to delineate species through hierarchical clustering based on sequence similarity [85]. A lower ASAP score indicates a better partition of the data set.

#### 4.3.3. Genetic Diversity of Taxa and Populations

To assess the taxon genealogy evolutionary lineages of the analyzed individuals, haplotype networks were constructed based on the chloroplast and mitochondrial data sets using the median-joining approach available in PopArt [47]. Genetic relationships between samples at the taxon level were visualized using principal coordinate analysis (PCoA) based on K2P distances and divergence hotspot data in the ggplot2 R package. The Kimura two-parameter (K2P) model [86] with 1000 bootstrap replicates was used to quantify divergence between populations of the *Pinus mugo* complex, as implemented in MEGA11 [82]. Average K2P distances were calculated from pairwise comparisons of all sequences within and between populations.

## 5. Conclusions

Based on the obtained results, we can conclude that genome skimming is very useful for the analysis of closely related taxa, as in the case of the *Pinus mugo* complex, because it allows the generation of a large amount of sequence data. It allows the acquisition of many (sometimes difficult in traditional sequencing) regions of mitochondrial, chloroplast, and nuclear DNA. The ITS1 region for representatives of the *Pinus mugo* complex, fully sequenced for the first time, although very long, is not too variable and, similarly to the ITS2 region, does not allow for the effective delimitation of these closely related taxa. The most promising data set for further discrimination analysis of the *Pinus mugo* complex is the divergence hotspot data set, which is nearly twenty-times more variable than traditional core chloroplast or nuclear DNA barcodes. *Pinus* × *rhaetica* should not be considered a synonym for *Pinus uliginosa* and *Pinus rotundata* because it differs significantly genetically from these two taxa and most likely represents a fixed hybrid or introgressant between *Pinus sylvestris* and *Pinus mugo*, as some previous studies have postulated. *Pinus uliginosa* and *Pinus rotundata*, despite the different scientific names, origins, and population histories, seem to have a similar genetic background, which is perhaps shaped by environmental pressures and the adaptation to the specific habitat they occupy. *Pinus uncinata* represents a clearly distinct clade within the *Pinus mugo* complex in most of the applied genetic data sets and analytical methods. The analysis of the pattern of genetic differentiation at the population level allows for a clear distinction of populations and taxa of the *Pinus mugo* complex from *Pinus sylvestris* and *Pinus* × *rhaetica*.

## Figures and Tables

**Table 1:** Comparison of variability characteristics of seven data sets in the *Pinus mugo* complex.

Alignment	Data Set Code	Length (bp)	Variable Sites (% divergence)	Parsimony Informative Sites (%)
Plastid coding genes	A	45,467	75 (0.165%)	17 (0.037%)
Mitochondrial coding genes	B	8378	128 (1.528%)	68 (0.812%)
Plastid intergenic regions	C	7092	59 (0.832%)	33 (0.465%)
nrDNA cistron	D	7849	10 (0.127%)	2 (0.025%)
ITS	E	2881	3 (0.104%)	0 (0.000%)
*matK* + *rbcL*	F	2976	8 (0.269%)	1 (0.034%)
Divergence hotspot regions	G	1021	20 (1.959%)	15 (1.469%)

**Table 2:** Summary of the rate of taxa discrimination using seven data sets based on tree-based, distance-based, and ASAP methods in combined *Pinus mugo* complex taxa, *Pinus × rhaetica*, and *Pinus sylvestris*. *—Considering all individuals from one taxon in the same operational taxonomic unit (OTU).

Alignment	Data Set Code	Tree-Based Method	Distance-Based Method	ASAP *
Plastid coding genes	A	0/6 (0.00%)	0/6 (0.0%)	0/6 (0.00%)
Mitochondrial coding genes	B	2/6 (33.33%)	2/6 (33.33%)	0/6 (0.00%)
Plastid intergenic regions	C	2/6 (33.33%)	2/6 (33.33%)	0/6 (0.00%)
nr DNA cistron	D	1/6 (16.67%)	1/6 (16.67%)	0/6 (0.00%)
ITS	E	0/6 (0.00%)	0/6 (0.00%)	0/6 (0.00%)
*matK + rbcL*	F	0/6 (0.00%)	0/6 (0.00%)	0/6 (0.00%)
Divergence hotspot regions	G	3/6 (50.00%)	3/6 (50.0%)	1/6 (16.67%)

## Data Availability

The original contributions presented in the study are included in the article/Supplementary Materials. Further inquiries can be directed to the corresponding author/s.

## Supplementary Materials

The following supporting information can be downloaded at: https://www.mdpi.com/article/10.3390/ijms251810178/s1.
