# Supplementary material for: Exploring Taxonomic and Genetic Relationships in the Pinus mugo Complex Using Genome Skimming Data
Source: Int J Mol Sci. 2024 Sep 22;25(18):10178. doi: 10.3390/ijms251810178 (PMC11432513; doi:10.3390/ijms251810178)
Supplement: Supplementary file 1 [file ijms-25-10178-s001.zip › ijms-3192741-supplementary.pdf]

Table S1. Plant materials used in this study.

Taxon	Location	Latitude (N), Longitude (E)	Population code	Sample size (individual code)	Country	Voucher
Pinus mugo	Tatra National Park	49°14′30 N, 20°00′15 E	TM	2 (1-2)	Poland	POZG-V-0150537 POZG-V-0150538
Pinus mugo	Tatra National Park	49°16′06 N, 20°02′40E	TW	2 (3-4)	Poland	POZG-V-0150539 POZG-V-0150540
Pinus uliginosa	"Torfowisko pod Węglińcem" Nature Reserve	51°17′36 N, 15°13′37 E	WG	3 (5-7)	Poland	POZG-V-0150541 POZG-V-0150542 POZG-V-0150543
Pinus uliginosa	"Wielkie Torfowisko Batorowskie" Nature Reserve	50°27′30 N, 16°22′57 E	BA	2 (8-9)	Poland	POZG-V-0150544 POZG-V-0150545
Pinus × rhaetica	"Bór nad Czerwonem" Nature Reserve	49° 27′30N 20° 02′25 E	BC	3 (10-12)	Poland	POZG-V-0150546 POZG-V-0150547 POZG-V-0150548
Pinus rotundata	"Kirchspielwald-Ibacher Moos" Nature and Forest Reserve	47°43′38 N, 8°03′01 E	IB	3 (13-15)	Germany	POZG-V-0150549 POZG-V-0150550 POZG-V-0150551
Pinus rotundata	"Rotmeer" Nature Reserve	47°51′53 N, 8°06′00 E	RO	3 (16-18)	Germany	POZG-V-0150552 POZG-V-0150553 POZG-V-0150554
Pinus rotundata	"Steerenmoos", Fauna-flora-habitat area: "Valley of Schwarza, Mettma, Schlücht and the Steina"	47°48′21 N, 8°12′00 E	ST	3 (19-21)	Germany	POZG-V-0150555 POZG-V-0150556 POZG-V-0150557
Pinus rotundata	"Novohůrecká slat", Šumava National Park	49°09′18 N, 13°19′46 E	NH	3 (22-24)	Czech Republic	POZG-V-0150558 POZG-V-0150559 POZG-V-0150560
Pinus rotundata	"Červené blato" National Nature Reserve	48°51′26 N, 14°48′08 E	CB	3 (25-27)	Czech Republic	POZG-V-0150561 POZG-V-0150562 POZG-V-0150563
Pinus sylvestris	Dendrological Garden, University of Life Sciences	52°25′32 N, 16°53′39 E	US	1 (28)	Poland	POZG-V-0150564
Pinus sylvestris	Morasko, Poznań	52°28′02 N, 16°55′30 E	MO	2 (29-30)	Poland	POZG-V-0150565 POZG-V-0150566
Pinus uncinata	Dendrological Garden, University of Life Sciences, Poznań	52°25′32 N, 16°53′39 E	UU	2 (31-32)	Poland	POZG-V-0150567 POZG-V-0150568

Table S2. List of sequence names used in datasets A, B, C, G.

No.	Plastid coding genes (data set A)	Mitochondrial coding genes (data set B)	Intergenic regions of the plastid (data set C)	Divergence hotspot regions (data set G)
1	*accD*	*ATP4*	*accD-psaI*	*rps1*
2	*atpA*	*ATP6*	*rps2-atpI*	*rps2*
3	*atpB*	*ATP8*	*clpP-rps12*	*rps14*
4	*atpF*	*ATP9*	*petB-petD*	*ycf3-psaA*
5	*atpH*	*ccmB*	*psaM-trnS*	*trnE-clpP*
6	*atpI*	*ccmC*	*psbJ-petA*	
7	*ccsA*	*cox3*	*psbL-psbJ*	
8	*cemA*	*NAD3*	*trnS-psbZ*	
9	*chlB*	*NAD4L*	*atpB-rbcL*	
10	*chlL*	*NAD6*	*rpoB-trnC*	
11	*chlN*	*rpl5*	*rrn4.5-rrn5*	
12	*matK*	*rps1*	*trnC-petN*	
13	*petA*	*rps2*	*trnD-psbM*	
14	*petB*	*rps14*	*trnE-clpP*	
15	*petD*	*rps19*	*trnL-trnF*	
16	*petG*		*trnM-ndhC*	
17	*petN*		*trnS-psaM*	
18	*psaB*		*ycf3-psaA*	
19	*psaC*			
20	*psbB*			
21	*psbC*			
22	*psbD*			
23	*psbE*			
24	*psbF*			
25	*psbH*			
26	*psbI*			
27	*psbJ*			
28	*psbK*			
29	*psbL*			
30	*psbM*			
31	*psbN*			
32	*psbT*			
33	*psbZ*			
34	*rbcL*			
35	*rpl2*			
36	*rpl14*			
37	*rpl16*			
38	*rpl20*			
39	*rpl22*			
40	*rpl23*			
41	*rpl33*			
42	*rpl36*			
43	*rpoA*			
44	*rpoB*			
45	*rpoC1*			
46	*rpoC2*			
47	*rps2*			
48	*rps3*			
49	*rps4*			

50	*rps7*
51	*rps11*
52	*rps14*
53	*rps15*
54	*rps18*
55	*rps19*
56	*ycf3*
57	*ycf4*

Table S3. Comparison of characteristics of seven data sets in *Pinus sylvestris* and *Pinus* × *rhaetica.*

**Alignment**	**Data set code**	**Length (bp)**	**Variable sites (% divergence)**	**Parsimony informative sites (%)**
Plastid coding genes	A	45,467	11 (0.024%)	2 (0.004%)
Mitochondrial coding genes	B	8,378	33 (0.394%)	15 (0.179%)
Plastid intergenic regions	C	7,092	22 (0.310%)	19 (0.268%)
nr DNA cistron	D	7,849	5 (0.064%)	0 (0.000%)
ITS	E	2,881	0 (0.000%)	0 (0.000%)
matK + *rbcL*	F	2,976	0 (0.000%)	0 (0.000%)
Divergence hotspot regions	G	1,021	3 (0.294%)	2 (0.196%)

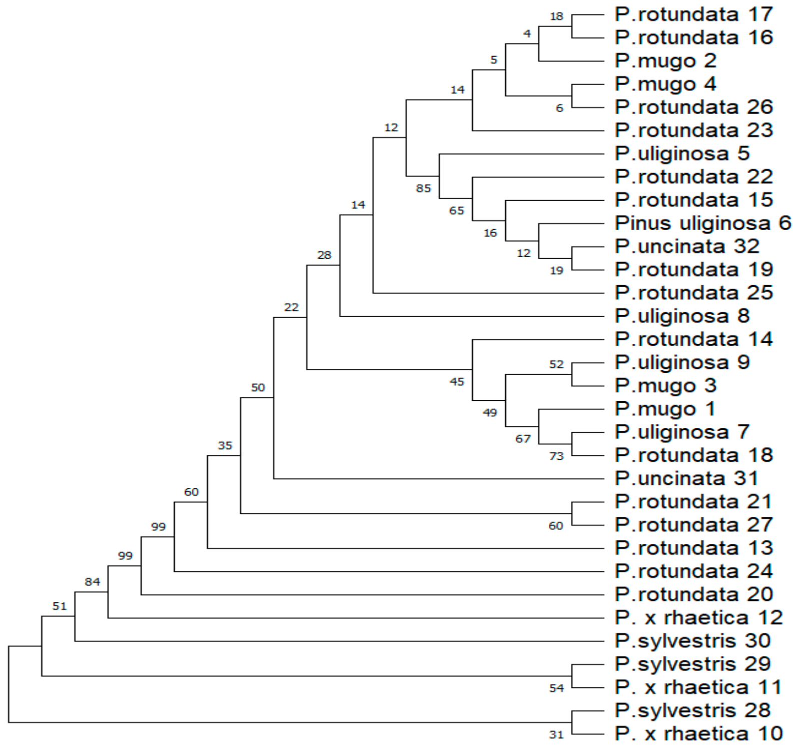

Figure S1. Maximum likelihood tree based on the plastid coding genes data set A.

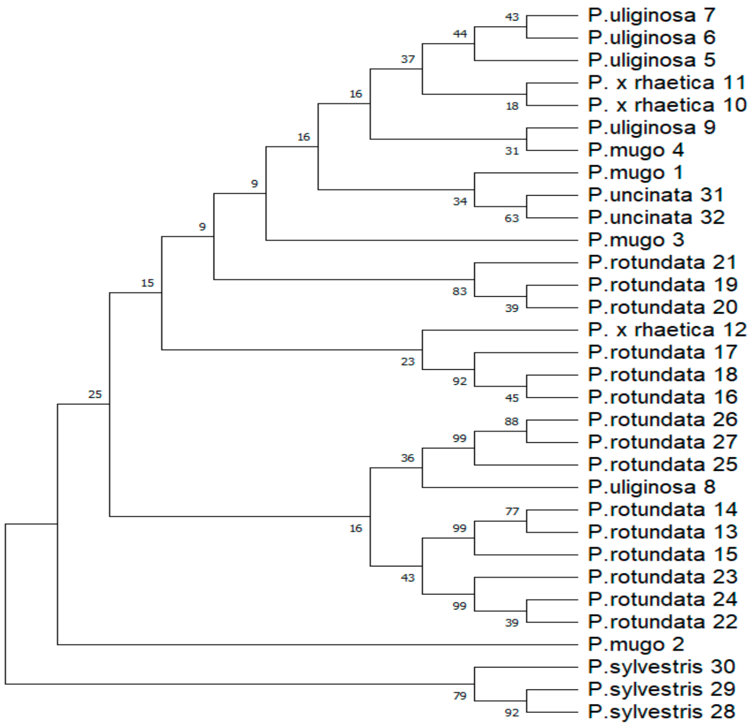

Figure S2. Maximum likelihood tree based on the mitochondrial coding genes data set B.

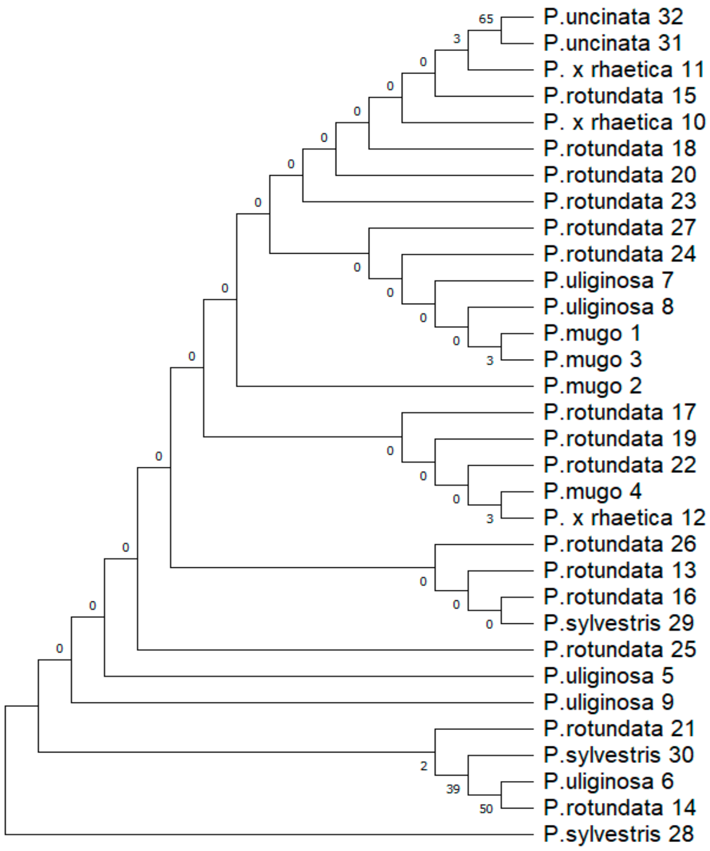

Figure S3. Maximum likelihood tree based on the nrDNA cistron data set D.

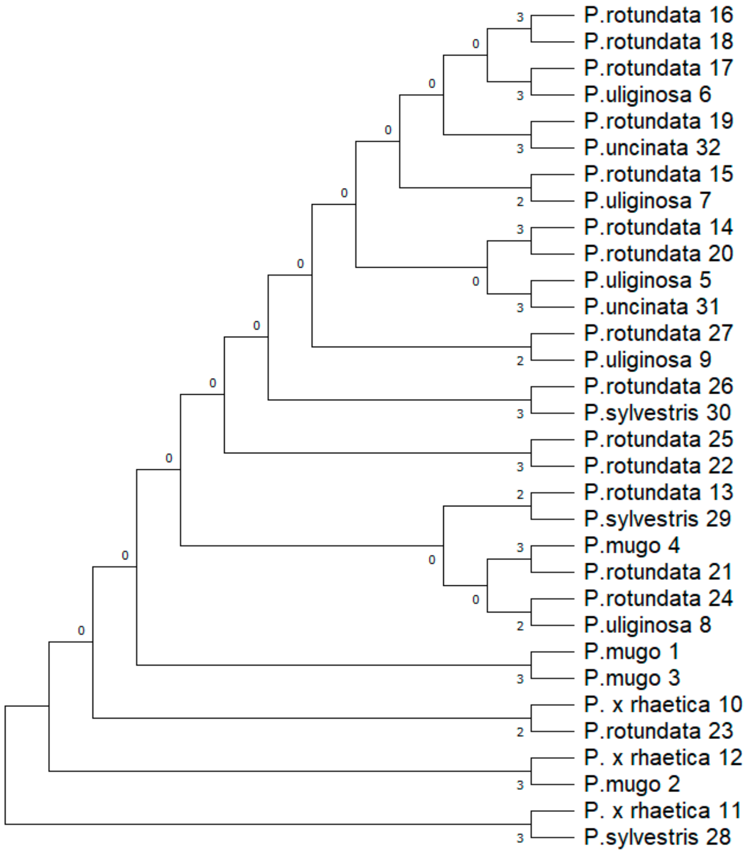

Figure S4. Maximum likelihood tree based on the ITS data set E.

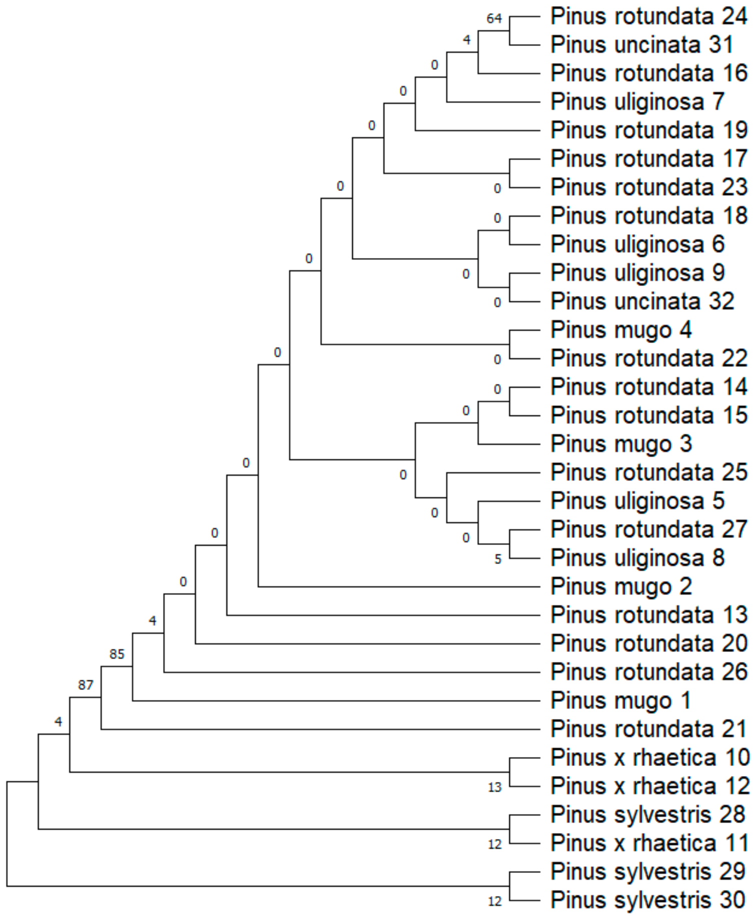

Figure S5. Maximum likelihood tree based on the *matK* + *rbcL* data set F.

Table S4. Minimum interspecific distance and maximum intraspecific distance for each taxa in the different data sets.

Taxon	Data set													
	A		B		C		D		E		F		G	
	min_inter	max_intra	min_inter	max_intra	min_inter	max_intra	min_inter	max_intra	min_inter	max_intra	min_inter	max_intra	min_inter	max_intra
Pinus mugo	0.00000	0.00009	0.00012	0.00143	0.00099	0.00227	0.00000	0.00013	0.00000	0.00000	0.00000	0.00000	0.00098	0.00294
Pinus uliginosa	0.00000	0.00015	0.00012	0.00227	0.00028	0.00170	0.00000	0.00013	0.00000	0.00000	0.00000	0.00000	0.00000	0.00591
Pinus × rhaetica	0.00007	0.00015	0.00071	0.00060	0.00199	0.00099	0.00000	0.00013	0.00000	0.00000	0.00000	0.00000	0.00196	0.00000
Pinus rotundata	0.00000	0.00106	0.00096	0.00491	0.00028	0.00355	0.00000	0.00064	0.00000	0.00000	0.00000	0.00236	0.00000	0.01285
Pinus sylvestris	0.00004	0.00009	0.00131	0.00215	0.00085	0.00114	0.00000	0.00051	0.00000	0.00000	0.00000	0.00000	0.00196	0.00195
Pinus uncinata	0.00004	0.00022	0.00060	0.00048	0.00029	0.00028	0.00040	0.00035	0.00000	0.00104	0.00000	0.00034	0.00295	0.00196

Note: the numbers colored by red represent the minimum interspecific distance larger than maximum intraspecific distance.

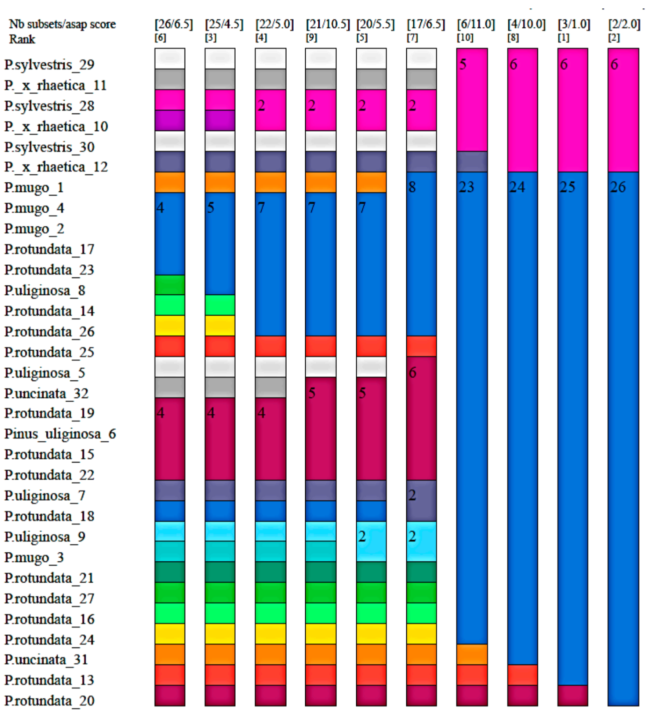

Figure S6. Species delimitation by ASAP analysis based on plastid coding genes. Graphical output showing the ten different delimitations; each column represents a partition, and the colors represent the OTUs. Every field contains the number of individuals. Above the colorful bars, the coefficient asap-score (the lower value) and number of species (the upper value) recognized for whole dataset are presented.

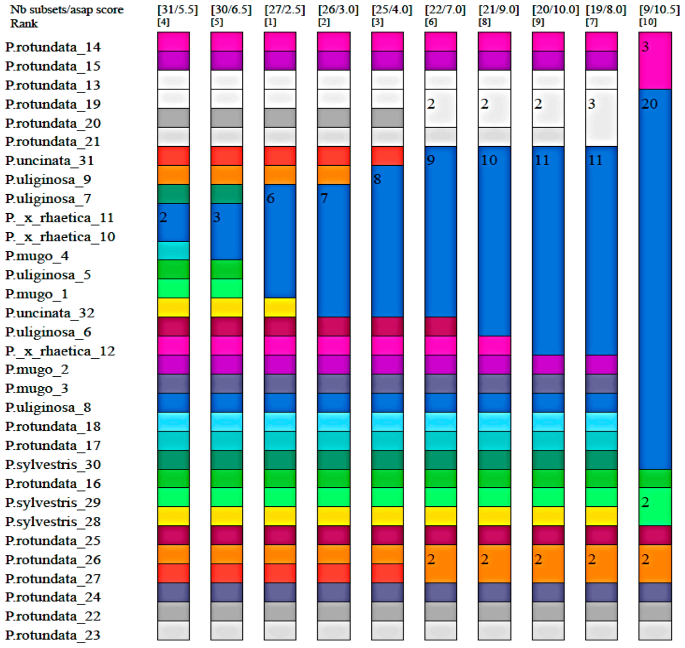

Figure S7. Species delimitation by ASAP analysis based on mitochondrial coding genes. Graphical output showing the ten different delimitations; each column represents a partition, and the colors represent the OTUs. Every field contains the number of individuals. Above the colorful bars, the coefficient asap-score (the lower value) and number of species (the upper value) recognized for whole dataset are presented.

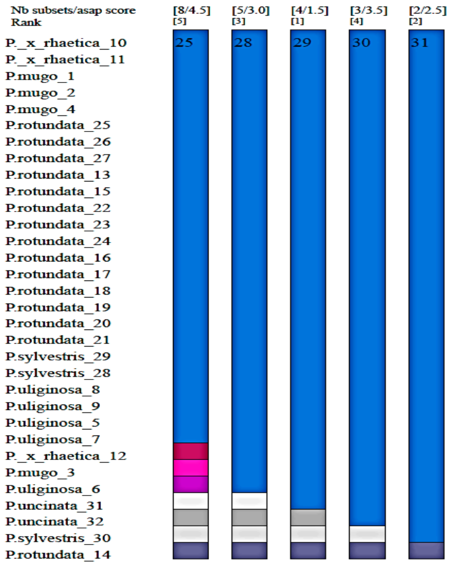

Figure S8. Species delimitation by ASAP analysis based on nrDNA cistron. Graphical output showing the ten different delimitations; each column represents a partition, and the colors represent the OTUs. Every field contains the number of individuals. Above the colorful bars, the coefficient asap-score (the lower value) and number of species (the upper value) recognized for whole dataset are presented.

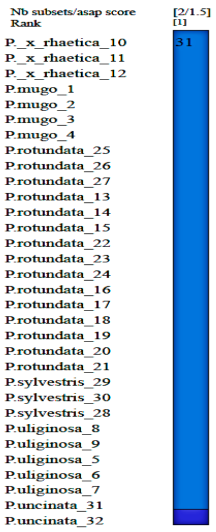

Figure S9. Species delimitation by ASAP analysis based on the internal transcribed spacer (ITS). Graphical output showing the ten different delimitations; each column represents a partition, and the colors represent the OTUs. Every field contains the number of individuals. Above the colorful bars, the coefficient asap-score (the lower value) and number of species (the upper value) recognized for whole dataset are presented.

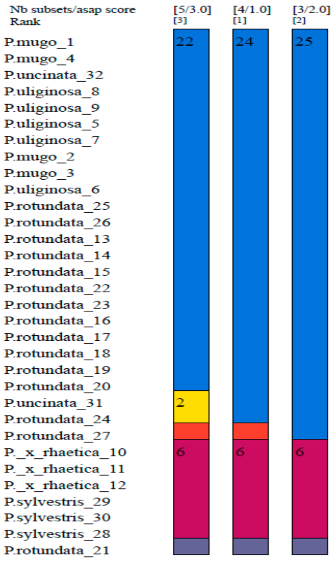

Figure S10. Species delimitation by ASAP analysis based on *matK* + *rbcL*. Graphical output showing the ten different delimitations; each column represents a partition, and the colors represent the OTUs. Every field contains the number of individuals. Above the colorful bars, the coefficient asap-score (the lower value) and number of species (the upper value) recognized for whole dataset are presented.

Table S5. Various haplotypes of *Pinus mugo* complex, *P. sylvestris and P.* × *rhaetica* taxa identified in the current study based on plastid coding genes.

**Haplotype**	**No. of sequences**	**Taxa**
Hap_1	1	*P. sylvestris* 29
Hap_2	1	*P.* × *rhaetica* 11
Hap_3	1	*P. sylvestris* 30
Hap_4	1	*P. sylvestris* 28
Hap_5	1	*P.* × *rhaetica* 10
Hap_6	1	*P.* × *rhaetica* 12
Hap_7	1	*P. mugo* 1
Hap_8	1	*P. uliginosa* 7
Hap_9	4	*P. mugo* 4, *P. mugo* 2, *P. rotundata* 17, *P. rotundata* 23
Hap_10	1	*P. uliginosa* 5
Hap_11	1	*P. uliginosa* 8
Hap_12	1	*P. uliginosa* 9
Hap_13	1	*P. uncinata* 32
Hap_14	1	*P. rotundata* 14
Hap_15	1	*P. rotundata* 26
Hap_16	1	*P. mugo* 3
Hap_17	1	*P. rotundata* 18
Hap_18	4	*P. rotundata* 19, *P. uliginosa* 6, *P. rotundata* 15, *P. rotundata* 22
Hap_19	1	*P. rotundata* 24
Hap_20	1	*P. rotundata* 21
Hap_21	1	*P. rotundata* 27
Hap_22	1	*P. rotundata* 16
Hap_23	1	*P. rotundata* 25
Hap_24	1	*P. uncinata* 31
Hap_25	1	*P. rotundata* 13
Hap_26	1	*P. rotundata* 20

Table S6. Various haplotypes of *Pinus mugo* complex*, P. sylvestris and P.* × *rhaetica* taxa identified in the current study based on mitochondrial coding genes.

**Haplotype**	**No. of sequences**	**Taxa**
Hap_1	1	*P. rotundata* 14
Hap_2	1	*P. rotundata* 13
Hap_3	1	*P. rotundata* 15
Hap_4	1	*P. rotundata* 19
Hap_5	1	*P. rotundata* 21
Hap_6	1	*P. rotundata* 20
Hap_7	1	*P. uliginosa* 7
Hap_8	1	*P. uliginosa* 5
Hap_9	2	*P.* × *rhaetica* 11, *P.* × *rhaetica* 10
Hap_10	1	*P. mugo* 1
Hap_11	1	*P. mugo* 4
Hap_12	1	*P. uncinata* 32
Hap_13	1	*P. uncinata* 31
Hap_14	1	*P. uliginosa* 9
Hap_15	1	*P. uliginosa* 6
Hap_16	1	*P.* × *rhaetica* 12
Hap_17	1	*P. mugo* 2
Hap_18	1	*P. mugo* 3
Hap_19	1	*P. uliginosa* 8
Hap_20	1	*P. sylvestris* 30
Hap_21	1	*P. rotundata* 18
Hap_22	1	*P. rotundata* 17
Hap_23	1	*P. rotundata* 16
Hap_24	1	*P. sylvestris* 28
Hap_25	1	*P. sylvestris* 29
Hap_26	1	*P. rotundata* 25
Hap_27	1	*P. rotundata* 26
Hap_28	1	*P. rotundata* 27
Hap_29	1	*P. rotundata* 24
Hap_30	1	*P. rotundata* 22

Hap_31	1	*P. rotundata* 23

Table S7. List of accession numbers of complete nrDNA cistron sequences used in this study.

No.	Taxa name	Accession number
1	*P.× rhaetica* 10	PQ094679
2	*P. × rhaetica* 11	PQ094680
3	*P. × rhaetica* 12	PQ094699
4	*P. mugo* 1	PQ094688
5	*P. mugo* 2	PQ094692
6	*P. mugo* 3	PQ094691
7	*P. mugo* 4	PQ094687
8	*P. rotundata* 25	PQ094668
9	*P. rotundata* 26	PQ094694
10	*P. rotundata* 27	PQ094667
11	*P. rotundata* 13	PQ094676
12	*P. rotundata* 14	PQ094697
13	*P. rotundata* 15	PQ094675
14	*P. rotundata* 22	PQ094688
15	*P. rotundata* 23	PQ094689
16	*P. rotundata* 24	PQ094695
17	*P. rotundata* 16	PQ094674
18	*P. rotundata* 17	PQ094690
19	*P. rotundata* 18	PQ094673
20	*P. rotundata* 19	PQ094696
21	*P. rotundata* 20	PQ094672
22	*P. rotundata* 21	PQ094671
23	*P. sylvestris* 29	PQ094677
24	*P. sylvestris* 30	PQ094689
25	*P. sylvestris* 28	PQ094678
26	*P. uliginosa* 8	PQ094682
27	*P. uliginosa* 9	PQ094681
28	*P. uliginosa* 5	PQ094686
29	*P. uliginosa* 6	PQ094693
30	*P. uliginosa* 7	PQ094685
31	*P. uncinata* 31	PQ094698
32	*P. uncinata* 32	PQ094700

Table S8. List of accession numbers of complete mitochondrial genes used in this study.

No.	Gene / Taxa	*ATP4*	*ATP6*	*ATP8*	*ATP9*	*ccmB*	*ccmC*	*COX3*	*NAD3*	*NAD4L*	*NAD6*	*rpl5*	*rps1*	*rps2*	*rps14*	*rps19*
1	*P. rotundata* 13	PQ255545	PQ255579	PQ255613	PQ255647	PQ342009	PQ342043	PQ342077	PQ342111	PQ342145	PQ342179	PQ342213	PQ342247	PQ342281	PQ342315	PQ342349
2	*P. rotundata* 14	PQ255546	PQ255580	PQ255614	PQ255648	PQ342010	PQ342044	PQ342078	PQ342112	PQ342146	PQ342180	PQ342214	PQ342248	PQ342282	PQ342316	PQ342350
3	*P. rotundata* 15	PQ255547	PQ255581	PQ255615	PQ255649	PQ342011	PQ342045	PQ342079	PQ342113	PQ342147	PQ342181	PQ342215	PQ342249	PQ342283	PQ342317	PQ342351
4	*P. rotundata* 19	PQ255548	PQ255582	PQ255616	PQ255650	PQ342012	PQ342046	PQ342080	PQ342114	PQ342148	PQ342182	PQ342216	PQ342250	PQ342284	PQ342318	PQ342352
5	*P. rotundata* 20	PQ255549	PQ255583	PQ255617	PQ255651	PQ342013	PQ342047	PQ342081	PQ342115	PQ342149	PQ342183	PQ342217	PQ342251	PQ342285	PQ342319	PQ342353
6	*P. rotundata* 21	PQ255550	PQ255584	PQ255618	PQ255652	PQ342014	PQ342048	PQ342082	PQ342116	PQ342150	PQ342184	PQ342218	PQ342252	PQ342286	PQ342320	PQ342354
7	*P. rotundata* 16	PQ255551	PQ255585	PQ255619	PQ255653	PQ342015	PQ342049	PQ342083	PQ342117	PQ342151	PQ342185	PQ342219	PQ342253	PQ342287	PQ342321	PQ342355
8	*P. rotundata* 17	PQ255552	PQ255586	PQ255620	PQ255654	PQ342016	PQ342050	PQ342084	PQ342118	PQ342152	PQ342186	PQ342220	PQ342254	PQ342288	PQ342322	PQ342356
9	*P. rotundata* 18	PQ255553	PQ255587	PQ255621	PQ255655	PQ342017	PQ342051	PQ342085	PQ342119	PQ342153	PQ342187	PQ342221	PQ342255	PQ342289	PQ342323	PQ342357
10	*P. rotundata* 25	PQ255554	PQ255588	PQ255622	PQ255656	PQ342018	PQ342052	PQ342086	PQ342120	PQ342154	PQ342188	PQ342222	PQ342256	PQ342290	PQ342324	PQ342358
11	*P. rotundata* 26	PQ255555	PQ255589	PQ255623	PQ255657	PQ342019	PQ342053	PQ342087	PQ342121	PQ342155	PQ342189	PQ342223	PQ342257	PQ342291	PQ342325	PQ342359
12	*P. rotundata* 27	PQ255556	PQ255590	PQ255624	PQ255658	PQ342020	PQ342054	PQ342088	PQ342122	PQ342156	PQ342190	PQ342224	PQ342258	PQ342292	PQ342326	PQ342360
13	*P. rotundata* 22	PQ255557	PQ255591	PQ255625	PQ255659	PQ342021	PQ342055	PQ342089	PQ342123	PQ342157	PQ342191	PQ342225	PQ342259	PQ342293	PQ342327	PQ342361
14	*P. rotundata* 23	PQ255558	PQ255592	PQ255626	PQ255660	PQ342022	PQ342056	PQ342090	PQ342124	PQ342158	PQ342192	PQ342226	PQ342260	PQ342294	PQ342328	PQ342362
15	*P. rotundata* 24	PQ255559	PQ255593	PQ255627	PQ255661	PQ342023	PQ342057	PQ342091	PQ342125	PQ342159	PQ342193	PQ342227	PQ342261	PQ342295	PQ342329	PQ342363
16	*P. uliginosa* 9	PQ255560	PQ255594	PQ255628	PQ255662	PQ342024	PQ342058	PQ342092	PQ342126	PQ342160	PQ342194	PQ342228	PQ342262	PQ342296	PQ342330	PQ342364
17	*P. uliginosa* 7	PQ255561	PQ255595	PQ255629	PQ255663	PQ342025	PQ342059	PQ342093	PQ342127	PQ342161	PQ342195	PQ342229	PQ342263	PQ342297	PQ342331	PQ342365
18	*P. uliginosa* 6	PQ255562	PQ255596	PQ255630	PQ255664	PQ342026	PQ342060	PQ342094	PQ342128	PQ342162	PQ342196	PQ342230	PQ342264	PQ342298	PQ342332	PQ342366
19	*P. uliginosa* 5	PQ255563	PQ255597	PQ255631	PQ255665	PQ342027	PQ342061	PQ342095	PQ342129	PQ342163	PQ342197	PQ342231	PQ342265	PQ342299	PQ342333	PQ342367
20	*P. uliginosa* 8	PQ255564	PQ255598	PQ255632	PQ255666	PQ342028	PQ342062	PQ342096	PQ342130	PQ342164	PQ342198	PQ342232	PQ342266	PQ342300	PQ342334	PQ342368
21	*P.* × *rhaetica* 10	PQ255565	PQ255599	PQ255633	PQ255667	PQ342029	PQ342063	PQ342097	PQ342131	PQ342165	PQ342199	PQ342233	PQ342267	PQ342301	PQ342335	PQ342369
22	*P.* × *rhaetica* 11	PQ255566	PQ255600	PQ255634	PQ255668	PQ342030	PQ342064	PQ342098	PQ342132	PQ342166	PQ342200	PQ342234	PQ342268	PQ342302	PQ342336	PQ342370
23	*P.* × *rhaetica* 12	PQ255567	PQ255601	PQ255635	PQ255669	PQ342031	PQ342065	PQ342099	PQ342133	PQ342167	PQ342201	PQ342235	PQ342269	PQ342303	PQ342337	PQ342371
24	*P. mugo* 1	PQ255568	PQ255602	PQ255636	PQ255670	PQ342032	PQ342066	PQ342100	PQ342134	PQ342168	PQ342202	PQ342236	PQ342270	PQ342304	PQ342338	PQ342372
25	*P. mugo* 2	PQ255569	PQ255603	PQ255637	PQ255671	PQ342033	PQ342067	PQ342101	PQ342135	PQ342169	PQ342203	PQ342237	PQ342271	PQ342305	PQ342339	PQ342373
26	*P. mugo* 3	PQ255570	PQ255604	PQ255638	PQ255672	PQ342034	PQ342068	PQ342102	PQ342136	PQ342170	PQ342204	PQ342238	PQ342272	PQ342306	PQ342340	PQ342374
27	*P. mugo* 4	PQ255571	PQ255605	PQ255639	PQ255673	PQ342035	PQ342069	PQ342105	PQ342139	PQ342171	PQ342205	PQ342239	PQ342273	PQ342307	PQ342343	PQ342375
28	*P. uncinata* 31	PQ255574	PQ255608	PQ255642	PQ255676	PQ342038	PQ342072	PQ342106	PQ342140	PQ342174	PQ342208	PQ342242	PQ342276	PQ342310	PQ342344	PQ342378
29	*P. uncinata* 32	PQ255575	PQ255609	PQ255643	PQ255677	PQ342039	PQ342073	PQ342107	PQ342141	PQ342175	PQ342209	PQ342243	PQ342277	PQ342311	PQ342345	PQ342379
30	*P. sylvestris* 30	PQ255576	PQ255610	PQ255644	PQ255678	PQ342040	PQ342074	PQ342108	PQ342142	PQ342176	PQ342210	PQ342244	PQ342278	PQ342312	PQ342346	PQ342380
31	*P. sylvestris* 28	PQ255577	PQ255611	PQ255645	PQ255679	PQ342041	PQ342075	PQ342109	PQ342143	PQ342177	PQ342211	PQ342245	PQ342279	PQ342313	PQ342347	PQ342381
32	*P. sylvestris* 29	PQ255578	PQ255612	PQ255646	PQ255680	PQ342042	PQ342076	PQ342110	PQ342144	PQ342178	PQ342212	PQ342246	PQ342280	PQ342314	PQ342348	PQ342382

Table S9. List of accession numbers of complete chloroplast genes used in this study.

No.	Taxa / Gene	*P.× rhaetica* 10	*P.× rhaetica* 11	*P.× rhaetica* 12	*P. mugo* 1	*P. mugo* 2	*P. mugo* 3	*P. mugo* 4	*P. uliginosa* 5	*P. uliginosa* 6	*P .uliginosa* 7	*P. uliginosa* 8	*P. uliginosa* 9	*P. uncinata* 31	*P. uncinata* 32	*P. sylvestris* 29	*P. sylvestris* 30
1	*accD*	PQ342383	PQ342384	PQ342385	PQ165116	PQ342386	PQ342387	MZ333466	PQ165118	PQ342388	PQ165119	PQ165117	MZ333465	PQ342389	MZ333464	PQ342390	PQ342391
2	*atpA*	PQ342708	PQ342709	PQ342710	PQ165116	PQ342711	PQ342712	MZ333466	PQ165118	PQ342713	PQ165119	PQ165117	MZ333465	PQ342714	MZ333464	PQ342715	PQ342716
3	*atpB*	PQ342408	PQ342409	PQ342410	PQ165116	PQ342411	PQ342412	MZ333466	PQ165118	PQ342413	PQ165119	PQ165117	MZ333465	PQ342414	MZ333464	PQ342415	PQ342416
4	*atpF*	PQ342733	PQ342734	PQ342735	PQ165116	PQ342736	PQ342737	MZ333466	PQ165118	PQ342738	PQ165119	PQ165117	MZ333465	PQ342739	MZ333464	PQ342740	PQ342741
5	*atpH*	PQ342758	PQ342759	PQ342760	PQ165116	PQ342761	PQ342762	MZ333466	PQ165118	PQ342763	PQ165119	PQ165117	MZ333465	PQ342764	MZ333464	PQ342765	PQ342766
6	*atpI*	PQ342558	PQ342559	PQ342560	PQ165116	PQ342561	PQ342562	MZ333466	PQ165118	PQ342563	PQ165119	PQ165117	MZ333465	PQ342564	MZ333464	PQ342565	PQ34256
7	*ccsA*	PQ342783	PQ342784	PQ342785	PQ165116	PQ342786	PQ342787	MZ333466	PQ165118	PQ342788	PQ165119	PQ165117	MZ333465	PQ342789	MZ333464	PQ342790	PQ342791
8	*cemA*	PQ342808	PQ342809	PQ342810	PQ165116	PQ342811	PQ342812	MZ333466	PQ165118	PQ342813	PQ165119	PQ165117	MZ333465	PQ342814	MZ333464	PQ342815	PQ342816
9	*chlB*	PQ342833	PQ342834	PQ342835	PQ165116	PQ342836	PQ342837	MZ333466	PQ165118	PQ342838	PQ165119	PQ165117	MZ333465	PQ342839	MZ333464	PQ342840	PQ342841
10	*chlL*	PQ342858	PQ342859	PQ342860	PQ165116	PQ342861	PQ342862	MZ333466	PQ165118	PQ342863	PQ165119	PQ165117	MZ333465	PQ342864	MZ333464	PQ342865	PQ342866
11	*chlN*	PQ342883	PQ342884	PQ342885	PQ165116	PQ342886	PQ342887	MZ333466	PQ165118	PQ342888	PQ165119	PQ165117	MZ333465	PQ342889	MZ333464	PQ342890	PQ342891
12	*matK*	PQ342908	PQ342909	PQ342910	PQ165116	PQ342911	PQ342912	MZ333466	PQ165118	PQ342913	PQ165119	PQ165117	MZ333465	PQ342914	MZ333464	PQ342915	PQ342916
13	*petA*	PQ342483	PQ342484	PQ342485	PQ165116	PQ342486	PQ342487	MZ333466	PQ165118	PQ342488	PQ165119	PQ165117	MZ333465	PQ342489	MZ333464	PQ342490	PQ342491
14	*petB*	PQ342433	PQ342434	PQ342435	PQ165116	PQ342436	PQ342437	MZ333466	PQ165118	PQ342438	PQ165119	PQ165117	MZ333465	PQ342439	MZ333464	PQ342440	PQ342441
15	*petD*	PQ342433	PQ342434	PQ342435	PQ165116	PQ342436	PQ342437	MZ333466	PQ165118	PQ342438	PQ165119	PQ165117	MZ333465	PQ342439	MZ333464	PQ342440	PQ342441
16	*petG*	PQ342933	PQ342934	PQ342935	PQ165116	PQ342936	PQ342937	MZ333466	PQ165118	PQ342938	PQ165119	PQ165117	MZ333465	PQ342939	MZ333464	PQ342940	PQ342941
17	*petN*	PQ342533	PQ342534	PQ342535	PQ165116	PQ342536	PQ342537	MZ333466	PQ165118	PQ342538	PQ165119	PQ165117	MZ333465	PQ342539	MZ333464	PQ342539	PQ342539
18	*psaB*	PQ342958	PQ342959	PQ342960	PQ165116	PQ342961	PQ342962	MZ333466	PQ165118	PQ342963	PQ165119	PQ165117	MZ333465	PQ342964	MZ333464	PQ342965	PQ342966
19	*psaC*	PQ342983	PQ342984	PQ342985	PQ165116	PQ342986	PQ342987	MZ333466	PQ165118	PQ342988	PQ165119	PQ165117	MZ333465	PQ342989	MZ333464	PQ342990	PQ342991
20	*psbB*	PQ343008	PQ343009	PQ343010	PQ165116	PQ343011	PQ343012	MZ333466	PQ165118	PQ343013	PQ165119	PQ165117	MZ333465	PQ343014	MZ333464	PQ343015	PQ343016
21	*psbC*	PQ343033	PQ343034	PQ343035	PQ165116	PQ343036	PQ343037	MZ333466	PQ165118	PQ343038	PQ165119	PQ165117	MZ333465	PQ343039	MZ333464	PQ343040	PQ343041
22	*psbD*	PQ343058	PQ343059	PQ343060	PQ165116	PQ343061	PQ343062	MZ333466	PQ165118	PQ343063	PQ165119	PQ165117	MZ333465	PQ343064	MZ333464	PQ343065	PQ343066
23	*psbE*	PQ343083	PQ343084	PQ343085	PQ165116	PQ343086	PQ343087	MZ333466	PQ165118	PQ343088	PQ165119	PQ165117	MZ333465	PQ343089	MZ333464	PQ343090	PQ343091
24	*psbF*	PQ343108	PQ343109	PQ343110	PQ165116	PQ343111	PQ343112	MZ333466	PQ165118	PQ343113	PQ165119	PQ165117	MZ333465	PQ343114	MZ333464	PQ343115	PQ343116
25	*psbH*	PQ343133	PQ343134	PQ343135	PQ165116	PQ343136	PQ343137	MZ333466	PQ165118	PQ343138	PQ165119	PQ165117	MZ333465	PQ343139	MZ333464	PQ343140	PQ343141
26	*psbI*	PQ343158	PQ343159	PQ343160	PQ165116	PQ343161	PQ343162	MZ333466	PQ165118	PQ343163	PQ165119	PQ165117	MZ333465	PQ343164	MZ333464	PQ343165	PQ343166
27	*psbJ*	PQ342483	PQ342484	PQ342485	PQ165116	PQ342486	PQ342487	MZ333466	PQ165118	PQ342488	PQ165119	PQ165117	MZ333465	PQ342489	MZ333464	PQ342490	PQ342491
28	*psbK*	PQ343183	PQ343184	PQ343185	PQ165116	PQ343186	PQ343187	MZ333466	PQ165118	PQ343188	PQ165119	PQ165117	MZ333465	PQ343189	MZ333464	PQ343190	PQ343191
29	*psbL*	PQ342483	PQ342484	PQ342485	PQ165116	PQ342486	PQ342487	MZ333466	PQ165118	PQ342488	PQ165119	PQ165117	MZ333465	PQ342489	MZ333464	PQ342490	PQ342491
30	*psbM*	PQ342608	PQ342609	PQ342610	PQ165116	PQ342611	PQ342612	MZ333466	PQ165118	PQ342613	PQ165119	PQ165117	MZ333465	PQ342614	MZ333464	PQ342615	PQ342616
31	*psbN*	PQ343208	PQ343209	PQ343210	PQ165116	PQ343211	PQ343212	MZ333466	PQ165118	PQ343213	PQ165119	PQ165117	MZ333465	PQ343214	MZ333464	PQ343215	PQ343216
32	*psbT*	PQ343233	PQ343234	PQ343235	PQ165116	PQ343236	PQ343237	MZ333466	PQ165118	PQ343238	PQ165119	PQ165117	MZ333465	PQ343239	MZ333464	PQ343240	PQ343241

33	*psbZ*	PQ342508	PQ342509	PQ342510	PQ165116	PQ342511	PQ342512	MZ333466	PQ165118	PQ342513	PQ165119	PQ165117	MZ333465	PQ342514	MZ333464	PQ342515	PQ342516
34	*rbcL*	PQ342408	PQ342409	PQ342410	PQ165116	PQ342411	PQ342412	MZ333466	PQ165118	PQ342413	PQ165119	PQ165117	MZ333465	PQ342414	MZ333464	PQ342415	PQ342416
35	*rpl2*	PQ343258	PQ343259	PQ343260	PQ165116	PQ343261	PQ343262	MZ333466	PQ165118	PQ343263	PQ165119	PQ165117	MZ333465	PQ343264	MZ333464	PQ343265	PQ343266
36	*rpl14*	PQ343283	PQ343284	PQ343285	PQ165116	PQ343286	PQ343287	MZ333466	PQ165118	PQ343288	PQ165119	PQ165117	MZ333465	PQ343289	MZ333464	PQ343290	PQ343291
37	*rpl16*	PQ343308	PQ343309	PQ343310	PQ165116	PQ343311	PQ343312	MZ333466	PQ165118	PQ343313	PQ165119	PQ165117	MZ333465	PQ343314	MZ333464	PQ343315	PQ343316
38	*rpl20*	PQ343333	PQ343334	PQ343335	PQ165116	PQ343336	PQ343337	MZ333466	PQ165118	PQ343338	PQ165119	PQ165117	MZ333465	PQ343339	MZ333464	PQ343340	PQ343341
39	*rpl22*	PQ343358	PQ343359	PQ343360	PQ165116	PQ343361	PQ343362	MZ333466	PQ165118	PQ343363	PQ165119	PQ165117	MZ333465	PQ343364	MZ333464	PQ343365	PQ343366
40	*rpl23*	PQ343383	PQ343384	PQ343385	PQ165116	PQ343386	PQ343387	MZ333466	PQ165118	PQ343388	PQ165119	PQ165117	MZ333465	PQ343389	MZ333464	PQ343390	PQ343391
41	*rpl33*	PQ343408	PQ343409	PQ343410	PQ165116	PQ343411	PQ343412	MZ333466	PQ165118	PQ343413	PQ165119	PQ165117	MZ333465	PQ343414	MZ333464	PQ343415	PQ343416
42	*rpl36*	PQ343433	PQ343434	PQ343435	PQ165116	PQ343436	PQ343437	MZ333466	PQ165118	PQ343438	PQ165119	PQ165117	MZ333465	PQ343439	MZ333464	PQ343440	PQ343441
43	*rpoA*	PQ343458	PQ343459	PQ343460	PQ165116	PQ343461	PQ343462	MZ333466	PQ165118	PQ343463	PQ165119	PQ165117	MZ333465	PQ343464	MZ333464	PQ343465	PQ343466
44	*rpoB*	PQ342533	PQ342534	PQ342535	PQ165116	PQ342536	PQ342537	MZ333466	PQ165118	PQ342538	PQ165119	PQ165117	MZ333465	PQ342539	MZ333464	PQ342539	PQ342539
45	*rpoC1*	PQ343483	PQ343484	PQ343485	PQ165116	PQ343486	PQ343487	MZ333466	PQ165118	PQ343488	PQ165119	PQ165117	MZ333465	PQ343489	MZ333464	PQ343490	PQ343491
46	*rpoC2*	PQ343508	PQ343509	PQ343510	PQ165116	PQ343511	PQ343512	MZ333466	PQ165118	PQ343513	PQ165119	PQ165117	MZ333465	PQ343514	MZ333464	PQ343515	PQ343516
47	*rps2*	PQ342558	PQ342559	PQ342560	PQ165116	PQ342561	PQ342562	MZ333466	PQ165118	PQ342563	PQ165119	PQ165117	MZ333465	PQ342564	MZ333464	PQ342565	PQ34256
48	*rps3*	PQ343533	PQ343534	PQ343535	PQ165116	PQ343536	PQ343537	MZ333466	PQ165118	PQ343538	PQ165119	PQ165117	MZ333465	PQ343539	MZ333464	PQ343540	PQ343541
49	*rps4*	PQ343558	PQ343559	PQ343560	PQ165116	PQ343561	PQ343562	MZ333466	PQ165118	PQ343563	PQ165119	PQ165117	MZ333465	PQ343564	MZ333464	PQ343565	PQ343566
50	*rps7*	PQ343583	PQ343584	PQ343585	PQ165116	PQ343586	PQ343587	MZ333466	PQ165118	PQ343588	PQ165119	PQ165117	MZ333465	PQ343589	MZ333464	PQ343590	PQ343591
51	*rps11*	PQ343608	PQ343609	PQ343610	PQ165116	PQ343611	PQ343612	MZ333466	PQ165118	PQ343613	PQ165119	PQ165117	MZ333465	PQ343614	MZ333464	PQ343615	PQ343616
52	*rps14*	PQ343633	PQ343634	PQ343635	PQ165116	PQ343636	PQ343637	MZ333466	PQ165118	PQ343638	PQ165119	PQ165117	MZ333465	PQ343639	MZ333464	PQ343640	PQ343641
53	*rps15*	PQ343658	PQ343659	PQ343660	PQ165116	PQ343661	PQ343662	MZ333466	PQ165118	PQ343663	PQ165119	PQ165117	MZ333465	PQ343664	MZ333464	PQ343665	PQ343666
54	*rps18*	PQ343683	PQ343684	PQ343685	PQ165116	PQ343686	PQ343687	MZ333466	PQ165118	PQ343688	PQ165119	PQ165117	MZ333465	PQ343689	MZ333464	PQ343690	PQ343691
55	*rps19*	PQ343708	PQ343709	PQ343710	PQ165116	PQ343711	PQ343712	MZ333466	PQ165118	PQ343713	PQ165119	PQ165117	MZ333465	PQ343714	MZ333464	PQ343715	PQ343716
56	*ycf3*	PQ342658	PQ342659	PQ342660	PQ165116	PQ342661	PQ342662	MZ333466	PQ165118	PQ342663	PQ165119	PQ165117	MZ333465	PQ342664	MZ333464	PQ342665	PQ342666
57	*ycf4*	PQ343733	PQ343734	PQ343735	PQ165116	PQ343736	PQ343737	MZ333466	PQ165118	PQ343738	PQ165119	PQ165117	MZ333465	PQ343739	MZ333464	PQ343740	PQ343741

No.	Taxa / Gene	*P. sylvestris* 28	*P. rotundata* 25	*P. rotundata* 26	*P. rotundata* 27	*P. rotundata* 13	*P. rotundata* 14	*P. rotundata* 15	*P. rotundata* 22	*P. rotundata* 23	*P. rotundata* 24	*P. rotundata* 16	*P. rotundata* 17	*P. rotundata* 18	*P. rotundata* 19	*P. rotundata* 20	*P. rotundata* 21
1	*accD*	PQ342392	PQ342393	PQ342394	PQ342395	PQ342396	PQ342397	PQ342398	PQ342399	PQ342400	PQ342401	PQ342402	PQ342403	PQ342404	PQ342405	PQ342406	PQ342407
2	*atpA*	PQ342717	PQ342718	PQ342719	PQ342720	PQ342721	PQ342722	PQ342723	PQ342724	PQ342725	PQ342726	PQ342727	PQ342728	PQ342729	PQ342730	PQ342731	PQ3427232
3	*atpB*	PQ342417	PQ342418	PQ342419	PQ342420	PQ342421	PQ342422	PQ342423	PQ342424	PQ342425	PQ342426	PQ342427	PQ342428	PQ342429	PQ342430	PQ342431	PQ342432
4	*atpF*	PQ342742	PQ342743	PQ342744	PQ342745	PQ342746	PQ342747	PQ342748	PQ342749	PQ342750	PQ342751	PQ342752	PQ342753	PQ342754	PQ342755	PQ342756	PQ342757
5	*atpH*	PQ342767	PQ342768	PQ342769	PQ342770	PQ342771	PQ342772	PQ342773	PQ342774	PQ342775	PQ342776	PQ342777	PQ342778	PQ342779	PQ342780	PQ342781	PQ342782
6	*atpI*	PQ342567	PQ342568	PQ342569	PQ342570	PQ342571	PQ342572	PQ342573	PQ342574	PQ342575	PQ342576	PQ342577	PQ342578	PQ342579	PQ342580	PQ342581	PQ342582
7	*ccsA*	PQ342792	PQ342793	PQ342794	PQ342795	PQ342796	PQ342797	PQ342798	PQ342799	PQ342800	PQ342801	PQ342802	PQ342803	PQ342804	PQ342805	PQ342806	PQ342807

8	*cemA*	PQ342817	PQ342818	PQ342819	PQ342820	PQ342821	PQ342822	PQ342823	PQ342824	PQ342825	PQ342826	PQ342827	PQ342828	PQ342829	PQ342830	PQ342831	PQ342832
9	*chlB*	PQ342842	PQ342843	PQ342844	PQ342845	PQ342846	PQ342847	PQ342848	PQ342849	PQ342850	PQ342851	PQ342852	PQ342853	PQ342854	PQ342855	PQ342856	PQ342857
10	*chlL*	PQ342867	PQ342868	PQ342869	PQ342870	PQ342871	PQ342872	PQ342873	PQ342874	PQ342875	PQ342876	PQ342877	PQ342878	PQ342879	PQ342880	PQ342881	PQ342882
11	*chlN*	PQ342892	PQ342893	PQ342894	PQ342895	PQ342896	PQ342897	PQ342898	PQ342899	PQ342900	PQ342901	PQ342902	PQ342903	PQ342904	PQ342905	PQ342906	PQ342907
12	*matK*	PQ342917	PQ342918	PQ342919	PQ342920	PQ342921	PQ342922	PQ342923	PQ342924	PQ342925	PQ342926	PQ342927	PQ342928	PQ342929	PQ342930	PQ342931	PQ342932
13	*petA*	PQ342492	PQ342493	PQ342494	PQ342495	PQ342496	PQ342497	PQ342498	PQ342499	PQ342500	PQ342501	PQ342502	PQ342503	PQ342504	PQ342505	PQ342506	PQ342507
14	*petB*	PQ342442	PQ342443	PQ342444	PQ342445	PQ342446	PQ342447	PQ342448	PQ342449	PQ342450	PQ342451	PQ342452	PQ342453	PQ342454	PQ342455	PQ342456	PQ342457
15	*petD*	PQ342442	PQ342443	PQ342444	PQ342445	PQ342446	PQ342447	PQ342448	PQ342449	PQ342450	PQ342451	PQ342452	PQ342453	PQ342454	PQ342455	PQ342456	PQ342457
16	*petG*	PQ342942	PQ342943	PQ342944	PQ342945	PQ342946	PQ342947	PQ342948	PQ342949	PQ342950	PQ342951	PQ342952	PQ342953	PQ342954	PQ342955	PQ342956	PQ342957
17	*petN*	PQ342542	PQ342543	PQ342544	PQ342545	PQ342546	PQ342547	PQ342548	PQ342549	PQ342550	PQ342551	PQ342552	PQ342553	PQ342554	PQ342555	PQ342556	PQ342557
18	*psaB*	PQ342967	PQ342968	PQ342969	PQ342970	PQ342971	PQ342972	PQ342973	PQ342974	PQ342975	PQ342976	PQ342977	PQ342978	PQ342979	PQ342980	PQ342981	PQ342982
19	*psaC*	PQ342992	PQ342993	PQ342994	PQ342995	PQ342996	PQ342997	PQ342998	PQ342999	PQ343000	PQ343001	PQ343002	PQ343003	PQ343004	PQ343005	PQ343006	PQ343007
20	*psbB*	PQ343017	PQ343018	PQ343019	PQ343020	PQ343021	PQ343022	PQ343023	PQ343024	PQ343025	PQ343026	PQ343027	PQ343028	PQ343029	PQ343030	PQ343031	PQ343032
21	*psbC*	PQ343042	PQ343043	PQ343044	PQ343045	PQ343046	PQ343047	PQ343048	PQ343049	PQ343050	PQ343051	PQ343052	PQ343053	PQ343054	PQ343055	PQ343056	PQ343057
22	*psbD*	PQ343067	PQ343068	PQ343069	PQ343070	PQ343071	PQ343072	PQ343073	PQ343074	PQ343075	PQ343076	PQ343077	PQ343078	PQ343079	PQ343080	PQ343081	PQ343082
23	*psbE*	PQ343092	PQ343093	PQ343094	PQ343095	PQ343096	PQ343097	PQ343098	PQ343099	PQ343100	PQ343101	PQ343102	PQ343103	PQ343104	PQ343105	PQ343106	PQ343107
24	*psbF*	PQ343117	PQ343118	PQ343119	PQ343120	PQ343121	PQ343122	PQ343123	PQ343124	PQ343125	PQ343126	PQ343127	PQ343128	PQ343129	PQ343130	PQ343131	PQ343132
25	*psbH*	PQ343142	PQ343144	PQ343145	PQ343146	PQ343147	PQ343148	PQ343149	PQ343150	PQ343151	PQ343151	PQ343152	PQ343153	PQ343154	PQ343155	PQ343156	PQ343157
26	*psbI*	PQ343167	PQ343168	PQ343169	PQ343170	PQ343171	PQ343172	PQ343173	PQ343174	PQ343175	PQ343176	PQ343177	PQ343178	PQ343179	PQ343180	PQ343181	PQ343182
27	*psbJ*	PQ342492	PQ342493	PQ342494	PQ342495	PQ342496	PQ342497	PQ342498	PQ342499	PQ342500	PQ342501	PQ342502	PQ342503	PQ342504	PQ342505	PQ342506	PQ342507
28	*psbK*	PQ343192	PQ343193	PQ343194	PQ343195	PQ343196	PQ343197	PQ343198	PQ343199	PQ343200	PQ343201	PQ343202	PQ343203	PQ343204	PQ343205	PQ343206	PQ343207
29	*psbL*	PQ342492	PQ342493	PQ342494	PQ342495	PQ342496	PQ342497	PQ342498	PQ342499	PQ342500	PQ342501	PQ342502	PQ342503	PQ342504	PQ342505	PQ342506	PQ342507
30	*psbM*	PQ342617	PQ342618	PQ342619	PQ342620	PQ342621	PQ342622	PQ342623	PQ342624	PQ342625	PQ342626	PQ342627	PQ342628	PQ342629	PQ342630	PQ342631	PQ342632
31	*psbN*	PQ343217	PQ343218	PQ343219	PQ343220	PQ343221	PQ343222	PQ343223	PQ343224	PQ343225	PQ343226	PQ343227	PQ343228	PQ343229	PQ343230	PQ343231	PQ3432232
32	*psbT*	PQ343242	PQ343243	PQ343244	PQ343245	PQ343246	PQ343247	PQ343248	PQ343249	PQ343250	PQ343251	PQ343252	PQ343253	PQ343254	PQ343255	PQ343256	PQ343257
33	*psbZ*	PQ342517	PQ342518	PQ342519	PQ342520	PQ342521	PQ342522	PQ342523	PQ342524	PQ342525	PQ342526	PQ342527	PQ342528	PQ342529	PQ342530	PQ342531	PQ342532
34	*rbcL*	PQ342417	PQ342418	PQ342419	PQ342420	PQ342421	PQ342422	PQ342423	PQ342424	PQ342425	PQ342426	PQ342427	PQ342428	PQ342429	PQ342430	PQ342431	PQ342432
35	*rpl2*	PQ343267	PQ343268	PQ343269	PQ343270	PQ343271	PQ343272	PQ343273	PQ343274	PQ343275	PQ343276	PQ343277	PQ343278	PQ343279	PQ343280	PQ343281	PQ343282
36	*rpl14*	PQ343292	PQ343293	PQ343294	PQ343295	PQ343296	PQ343297	PQ343298	PQ343299	PQ343300	PQ343301	PQ343302	PQ343303	PQ343304	PQ343305	PQ343306	PQ343307
37	*rpl16*	PQ343317	PQ343318	PQ343319	PQ343320	PQ343321	PQ343322	PQ343323	PQ343324	PQ343325	PQ343326	PQ343327	PQ343328	PQ343329	PQ343330	PQ343331	PQ343332
38	*rpl20*	PQ343342	PQ343343	PQ343344	PQ343345	PQ343346	PQ343347	PQ343348	PQ343349	PQ343350	PQ343351	PQ343352	PQ343353	PQ343354	PQ343355	PQ343356	PQ343357
39	*rpl22*	PQ343367	PQ343368	PQ343369	PQ343370	PQ343371	PQ343372	PQ343373	PQ343374	PQ343375	PQ343376	PQ343377	PQ343378	PQ343379	PQ343380	PQ343381	PQ34332
40	*rpl23*	PQ343392	PQ343393	PQ343394	PQ343395	PQ343396	PQ343397	PQ343398	PQ343399	PQ343400	PQ343401	PQ343402	PQ343403	PQ343404	PQ343405	PQ343406	PQ343407
41	*rpl33*	PQ343417	PQ343418	PQ343419	PQ343420	PQ343421	PQ343422	PQ343423	PQ343424	PQ343425	PQ343426	PQ343427	PQ343428	PQ343429	PQ343430	PQ343431	PQ343432
42	*rpl36*	PQ343442	PQ343443	PQ343444	PQ343445	PQ343446	PQ343447	PQ343448	PQ343449	PQ343450	PQ343451	PQ343452	PQ343453	PQ343454	PQ343455	PQ343456	PQ343457
43	*rpoA*	PQ343467	PQ343468	PQ343469	PQ343470	PQ343471	PQ343472	PQ343473	PQ343474	PQ343475	PQ343476	PQ343477	PQ343478	PQ343479	PQ343480	PQ343481	PQ343482
44	*rpoB*	PQ342542	PQ342543	PQ342544	PQ342545	PQ342546	PQ342547	PQ342548	PQ342549	PQ342550	PQ342551	PQ342552	PQ342553	PQ342554	PQ342555	PQ342556	PQ342557
45	*rpoC1*	PQ343492	PQ343493	PQ343494	PQ343495	PQ343496	PQ343497	PQ3434938	PQ343499	PQ343500	PQ343501	PQ343502	PQ343503	PQ343504	PQ343505	PQ343506	PQ343507
46	*rpoC2*	PQ343517	PQ343518	PQ343519	PQ343520	PQ343521	PQ343522	PQ343523	PQ343524	PQ343525	PQ343526	PQ343527	PQ343528	PQ343529	PQ343530	PQ343531	PQ343532
47	*rps2*	PQ342567	PQ342568	PQ342569	PQ342570	PQ342571	PQ342572	PQ342573	PQ342574	PQ342575	PQ342576	PQ342577	PQ342578	PQ342579	PQ342580	PQ342581	PQ342582

48	*rps3*	PQ343542	PQ343543	PQ343544	PQ343545	PQ343546	PQ343547	PQ343548	PQ343549	PQ343550	PQ343551	PQ343552	PQ343553	PQ343554	PQ343555	PQ343556	PQ343557
49	*rps4*	PQ343567	PQ343568	PQ343569	PQ343570	PQ343571	PQ343572	PQ343573	PQ343574	PQ343575	PQ343576	PQ343577	PQ343578	PQ343579	PQ343580	PQ343581	PQ343582
50	*rps7*	PQ343592	PQ343593	PQ343594	PQ343595	PQ343596	PQ343597	PQ343598	PQ343599	PQ343600	PQ343601	PQ343602	PQ343603	PQ343604	PQ343605	PQ343606	PQ343607
51	*rps11*	PQ343617	PQ343618	PQ343619	PQ343620	PQ343621	PQ343622	PQ343623	PQ343624	PQ343625	PQ343626	PQ343627	PQ343628	PQ343629	PQ343630	PQ343631	PQ343632
52	*rps14*	PQ343642	PQ343643	PQ343644	PQ343645	PQ343646	PQ343647	PQ343648	PQ343649	PQ343650	PQ343651	PQ343652	PQ343653	PQ343654	PQ343655	PQ343656	PQ343657
53	*rps15*	PQ343667	PQ343668	PQ343669	PQ343670	PQ343671	PQ343672	PQ343673	PQ343674	PQ343675	PQ343676	PQ343677	PQ343678	PQ343679	PQ343680	PQ343681	PQ343682
54	*rps18*	PQ343692	PQ343693	PQ343694	PQ343695	PQ343696	PQ343697	PQ343698	PQ343699	PQ343700	PQ343701	PQ343702	PQ343703	PQ343704	PQ343705	PQ343706	PQ343707
55	*rps19*	PQ343717	PQ343718	PQ343719	PQ343720	PQ343721	PQ343722	PQ343723	PQ343724	PQ343725	PQ343726	PQ343727	PQ343728	PQ343729	PQ343730	PQ343731	PQ343732
56	*ycf3*	PQ342667	PQ342668	PQ342669	PQ342670	PQ342671	PQ342672	PQ342673	PQ342674	PQ342675	PQ342676	PQ342677	PQ342678	PQ342679	PQ342680	PQ342681	PQ342682
57	*ycf4*	PQ343742	PQ343743	PQ343744	PQ343745	PQ343746	PQ343747	PQ343748	PQ343749	PQ343750	PQ343751	PQ343752	PQ343753	PQ343754	PQ343755	PQ343756	PQ343757

Table S10. List of accession numbers of plastid intergenic regions used in this study.

No.	Taxa / Intergenic spacer	*P.× rhaetica* 10	*P.× rhaetica* 11	*P.× rhaetica* 12	*P. mugo* 1	*P. mugo* 2	*P. mugo* 3	*P. mugo* 4	*P. uliginosa* 5	*P. uliginosa* 6	*P. uliginosa* 7	*P. uliginosa* 8	*P. uliginosa* 9	*P. uncinata* 31	*P. uncinata* 32	*P . sylvestris* 29	*P . sylvestris* 30
1	*accD-psaI*	PQ342383	PQ342384	PQ342385	PQ165116	PQ342386	PQ342387	MZ333466	PQ165118	PQ342388	PQ165119	PQ165117	MZ333465	PQ342389	MZ333464	PQ342390	PQ342391
2	*rps2-atpI*	PQ342558	PQ342559	PQ342560	PQ165116	PQ342561	PQ342562	MZ333466	PQ165118	PQ342563	PQ165119	PQ165117	MZ333465	PQ342564	MZ333464	PQ342565	PQ34256
3	*clpP-rps12*	PQ343758	PQ343759	PQ343760	PQ165116	PQ343761	PQ343762	MZ333466	PQ165118	PQ343763	PQ165119	PQ165117	MZ333465	PQ343764	MZ333464	PQ343765	PQ343766
4	*petB-petD*	PQ342433	PQ342434	PQ342435	PQ165116	PQ342436	PQ342437	MZ333466	PQ165118	PQ342438	PQ165119	PQ165117	MZ333465	PQ342439	MZ333464	PQ342440	PQ342441
5	*psaM-trnS*	PQ342458	PQ342459	PQ342460	PQ165116	PQ342461	PQ342462	MZ333466	PQ165118	PQ342463	PQ165119	PQ165117	MZ333465	PQ342464	MZ333464	PQ342465	PQ342466
6	*psbJ-petA*	PQ342483	PQ342484	PQ342485	PQ165116	PQ342486	PQ342487	MZ333466	PQ165118	PQ342488	PQ165119	PQ165117	MZ333465	PQ342489	MZ333464	PQ342490	PQ342491
7	*psbL-psbJ*	PQ342483	PQ342484	PQ342485	PQ165116	PQ342486	PQ342487	MZ333466	PQ165118	PQ342488	PQ165119	PQ165117	MZ333465	PQ342489	MZ333464	PQ342490	PQ342491
8	*trnS-psbZ*	PQ342508	PQ342509	PQ342510	PQ165116	PQ342511	PQ342512	MZ333466	PQ165118	PQ342513	PQ165119	PQ165117	MZ333465	PQ342514	MZ333464	PQ342515	PQ342516
9	*atpB-rbcL*	PQ342408	PQ342409	PQ342410	PQ165116	PQ342411	PQ342412	MZ333466	PQ165118	PQ342413	PQ165119	PQ165117	MZ333465	PQ342414	MZ333464	PQ342415	PQ342416
10	*rpoB-trnC*	PQ342533	PQ342534	PQ342535	PQ165116	PQ342536	PQ342537	MZ333466	PQ165118	PQ342538	PQ165119	PQ165117	MZ333465	PQ342539	MZ333464	PQ342539	PQ342539
11	*rrn4.5-rrn5*	PQ342583	PQ342584	PQ342585	PQ165116	PQ342586	PQ342587	MZ333466	PQ165118	PQ342588	PQ165119	PQ165117	MZ333465	PQ342589	MZ333464	PQ342590	PQ342591
12	*trnC-petN*	PQ342533	PQ342534	PQ342535	PQ165116	PQ342536	PQ342537	MZ333466	PQ165118	PQ342538	PQ165119	PQ165117	MZ333465	PQ342539	MZ333464	PQ342539	PQ342539
13	*trnD-psbM*	PQ342608	PQ342609	PQ342610	PQ165116	PQ342611	PQ342612	MZ333466	PQ165118	PQ342613	PQ165119	PQ165117	MZ333465	PQ342614	MZ333464	PQ342615	PQ342616
14	*trnE-clpP*	PQ343758	PQ343759	PQ343760	PQ165116	PQ343761	PQ343762	MZ333466	PQ165118	PQ343763	PQ165119	PQ165117	MZ333465	PQ343764	MZ333464	PQ343765	PQ343766
15	*trnL-trnF*	PQ343783	PQ343784	PQ343785	PQ165116	PQ343786	PQ343787	MZ333466	PQ165118	PQ343788	PQ165119	PQ165117	MZ333465	PQ343789	MZ333464	PQ343790	PQ343791
16	*trnM-ndhC*	PQ342633	PQ342634	PQ342635	PQ165116	PQ342636	PQ342637	MZ333466	PQ165118	PQ342638	PQ165119	PQ165117	MZ333465	PQ342639	MZ333464	PQ342640	PQ342641
17	*trnS-psaM*	PQ342683	PQ342684	PQ342685	PQ165116	PQ342686	PQ342687	MZ333466	PQ165118	PQ342688	PQ165119	PQ165117	MZ333465	PQ342689	MZ333464	PQ342690	PQ342691
18	*ycf3-psaA*	PQ342658	PQ342659	PQ342660	PQ165116	PQ342661	PQ342662	MZ333466	PQ165118	PQ342663	PQ165119	PQ165117	MZ333465	PQ342664	MZ333464	PQ342665	PQ342666

No.	Taxa / Intergenic spacer	*P. sylvestris* 28	*P. rotundata* 25	*P. rotundata* 26	*P. rotundata* 27	*P. rotundata* 13	*P. rotundata* 14	*P. rotundata* 15	*P. rotundata* 22	*P. rotundata* 23	*P. rotundata* 24	*P. rotundata* 16	*P. rotundata* 17	*P. rotundata* 18	*P. rotundata* 19	*P. rotundata* 20	*P. rotundata* 21
1	*accD-psaI*	PQ342392	PQ342393	PQ342394	PQ342395	PQ342396	PQ342397	PQ342398	PQ342399	PQ342400	PQ342401	PQ342402	PQ342403	PQ342404	PQ342405	PQ342406	PQ342407
2	*rps2-atpI*	PQ342567	PQ342568	PQ342569	PQ342570	PQ342571	PQ342572	PQ342573	PQ342574	PQ342575	PQ342576	PQ342577	PQ342578	PQ342579	PQ342580	PQ342581	PQ342582
3	*clpP-rps12*	PQ343767	PQ343768	PQ343769	PQ343770	PQ343771	PQ343772	PQ343773	PQ343774	PQ343775	PQ343776	PQ343777	PQ343778	PQ343779	PQ343780	PQ343781	PQ343782

4	*petB-petD*	PQ342442	PQ342443	PQ342444	PQ342445	PQ342446	PQ342447	PQ342448	PQ342449	PQ342450	PQ342451	PQ342452	PQ342453	PQ342454	PQ342455	PQ342456	PQ342457
5	*psaM-trnS*	PQ342467	PQ342468	PQ342469	PQ342470	PQ342471	PQ342472	PQ342473	PQ342474	PQ342475	PQ342476	PQ342477	PQ342478	PQ342479	PQ342480	PQ342481	PQ342482
6	*psbJ-petA*	PQ342492	PQ342493	PQ342494	PQ342495	PQ342496	PQ342497	PQ342498	PQ342499	PQ342500	PQ342501	PQ342502	PQ342503	PQ342504	PQ342505	PQ342506	PQ342507
7	*psbL-psbJ*	PQ342492	PQ342493	PQ342494	PQ342495	PQ342496	PQ342497	PQ342498	PQ342499	PQ342500	PQ342501	PQ342502	PQ342503	PQ342504	PQ342505	PQ342506	PQ342507
8	*trnS-psbZ*	PQ342517	PQ342518	PQ342519	PQ342520	PQ342521	PQ342522	PQ342523	PQ342524	PQ342525	PQ342526	PQ342527	PQ342528	PQ342529	PQ342530	PQ342531	PQ342532
9	*atpB-rbcL*	PQ342417	PQ342418	PQ342419	PQ342420	PQ342421	PQ342422	PQ342423	PQ342424	PQ342425	PQ342426	PQ342427	PQ342428	PQ342429	PQ342430	PQ342431	PQ342432
10	*rpoB-trnC*	PQ342542	PQ342543	PQ342544	PQ342545	PQ342546	PQ342547	PQ342548	PQ342549	PQ342550	PQ342551	PQ342552	PQ342553	PQ342554	PQ342555	PQ342556	PQ342557
11	*rrn4.5-rrn5*	PQ342592	PQ342593	PQ342594	PQ342595	PQ342596	PQ342597	PQ342598	PQ342599	PQ342600	PQ342601	PQ342602	PQ342603	PQ342604	PQ342605	PQ342606	PQ342607
12	*trnC-petN*	PQ342542	PQ342543	PQ342544	PQ342545	PQ342546	PQ342547	PQ342548	PQ342549	PQ342550	PQ342551	PQ342552	PQ342553	PQ342554	PQ342555	PQ342556	PQ342557
13	*trnD-psbM*	PQ342617	PQ342618	PQ342619	PQ342620	PQ342621	PQ342622	PQ342623	PQ342624	PQ342625	PQ342626	PQ342627	PQ342628	PQ342629	PQ342630	PQ342631	PQ342632
14	*trnE-clpP*	PQ343767	PQ343768	PQ343769	PQ343770	PQ343771	PQ343772	PQ343773	PQ343774	PQ343775	PQ343776	PQ343777	PQ343778	PQ343779	PQ343780	PQ343781	PQ343782
15	*trnL-trnF*	PQ343792	PQ343793	PQ343794	PQ343795	PQ343796	PQ343797	PQ343798	PQ343799	PQ343800	PQ343801	PQ343802	PQ343803	PQ343804	PQ343805	PQ343806	PQ343807
16	*trnM-ndhC*	PQ342642	PQ342643	PQ342644	PQ342645	PQ342646	PQ342647	PQ342648	PQ342649	PQ342650	PQ342651	PQ342652	PQ342653	PQ342654	PQ342655	PQ342656	PQ342657
17	*trnS-psaM*	PQ342692	PQ342693	PQ342694	PQ342695	PQ342696	PQ342697	PQ342698	PQ342699	PQ342700	PQ342701	PQ342702	PQ342703	PQ342704	PQ342705	PQ342706	PQ342707
18	*ycf3-psaA*	PQ342667	PQ342668	PQ342669	PQ342670	PQ342671	PQ342672	PQ342673	PQ342674	PQ342675	PQ342676	PQ342677	PQ342678	PQ342679	PQ342680	PQ342681	PQ342682
